# Combining predictive coding and neural oscillations enables online syllable recognition in natural speech

**DOI:** 10.1038/s41467-020-16956-5

**Published:** 2020-06-19

**Authors:** Sevada Hovsepyan, Itsaso Olasagasti, Anne-Lise Giraud

**Affiliations:** Department of Basic Neurosciences, University of Geneva, Biotech Campus, 9 Chemin des Mines, C.P. 87, 1211 Genève, Switzerland

**Keywords:** Computational neuroscience, Sensory processing

## Abstract

On-line comprehension of natural speech requires segmenting the acoustic stream into discrete linguistic elements. This process is argued to rely on theta-gamma oscillation coupling, which can parse syllables and encode them in decipherable neural activity. Speech comprehension also strongly depends on contextual cues that help predicting speech structure and content. To explore the effects of theta-gamma coupling on bottom-up/top-down dynamics during on-line syllable identification, we designed a computational model (Precoss—predictive coding and oscillations for speech) that can recognise syllable sequences in continuous speech. The model uses predictions from internal spectro-temporal representations of syllables and theta oscillations to signal syllable onsets and duration. Syllable recognition is best when theta-gamma coupling is used to temporally align spectro-temporal predictions with the acoustic input. This neurocomputational modelling work demonstrates that the notions of predictive coding and neural oscillations can be brought together to account for on-line dynamic sensory processing.

## Abstract (web summary)

The authors present a model to parse and recognise syllables on-line in natural speech sentences that combine predictive coding and neural oscillations. They use simulations from different versions of the model to establish the importance of both theta-gamma coupling and the reset of accumulated evidence in continuous speech processing.

## Introduction

Neural oscillations are involved in many different cognitive operations^1–3^, and considering their cross-frequency coupling permits to more closely approach their function, e.g., perception, memory, attention, etc.^4^. In the domain of natural speech recognition, an important role has been assigned to the coupling of theta and gamma oscillations^5–7^, as it permits to hierarchically coordinate the encoding of phonemes within syllables, without prior knowledge of their duration and temporal occurrence, i.e. in a purely bottom-up online manner^8^.

Natural speech recognition also strongly relies on contextual cues to anticipate the content and temporal structure of the speech signal^9–11^. Recent studies underline the importance of top-down predictive mechanisms during continuous speech perception and relate them to another range of oscillatory activity, the low-beta band^12–17^. Predictive coding^18–21^, on the other hand, offers a theory of brain function that, in common with Analysis-by-Synthesis^22,23^ and the Bayesian Brain hypothesis^24^, relies on the agent having an internal model of how sensory signals are generated from their underlying hidden causes. Predictive coding also provides a message passing scheme in which top-down predictions and bottom-up prediction errors work together to identify the hidden causes of sensory signals. It therefore incorporates the contextual and prior knowledge which are invoked as critical in speech processing^25^.

Bottom-up and top-down approaches of speech processing both find support in theoretical studies. A neurocomputational model involving the coupling of realistic theta and gamma excitatory/inhibitory networks was able to pre-process speech in such a way that it could then be correctly decoded^8^. This model aimed at understanding the computational potential of biophysically realistic oscillatory neural processes rather than simply fitting existing data. A radically different model, solely based on predictive coding, could faithfully recognise isolated speech items (such as words, or full sentences when considered as a single speech item)^26^. Although both approaches intend to describe speech perception, one model focused on the on-line parsing aspect of speech processing, and the other on the recognition of isolated speech segments (no parsing needed). Combining the physiological notion of neural oscillations with the cognitive notion of predictive coding is appealing^27^ as it could broaden the capacity, improve performance, and enhance the biological realism of neurocomputational models of speech processing. More fundamentally, such an attempt offers the opportunity to explore the possible orchestration between two equally important neuroscientific levels of description, computational/algorithmic for analysis-by-synthesis and algorithmic/implementational for neural oscillations^28^.

In this study, we addressed whether a predictive coding speech recognition model could benefit from neural oscillation processes. We designed the Precoss  neurocomputational model based on the predictive coding framework in which we included theta and gamma oscillatory functions to deal with the continuous nature of natural speech. Although natural sentence comprehension involves many processing steps up to the syntax level, the parsing of syllables and their on-line recognition is a crucial step and a challenging issue, even for current automatic speech recognition (ASR) systems^29–31^. The specific goal of this modelling work was hence to address whether combining predictive coding and neural oscillations could be advantageous for on-line identification of the syllabic components of natural sentences. Specifically, we examined the possible mechanisms by which theta oscillations can interact with bottom-up and top-down information flows and assessed the effects of this interaction on the efficacy of the syllable decoding process. We show that on-line syllable identification from speech works best when theta-gamma coupling is combined with the internal knowledge about syllables’ spectral structure, and more broadly when continuous inferential processes are informed by dynamic oscillation-based cues.

## Results

### Precoss architecture and theta-gamma coupling

**Fig. 1:**
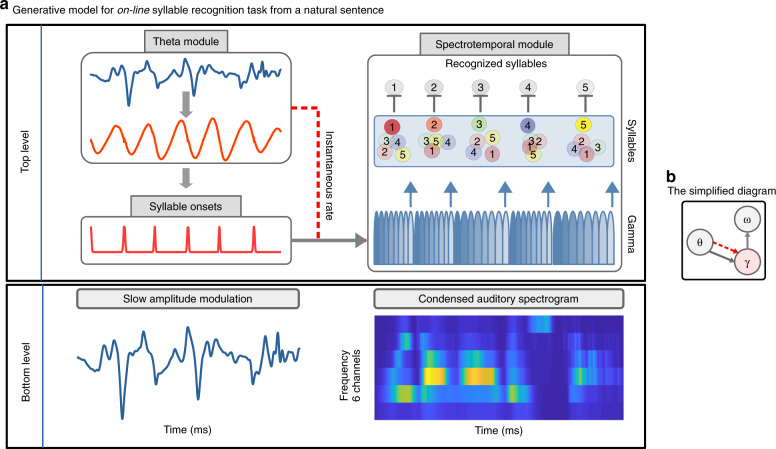
Model of on-line syllable parsing and identification from natural sentences—Precoss. **a** The bottom level encodes the dynamics in the input signal, which consists of two parts; the condensed auditory spectrogram^37^ (on the right) and the slow amplitude modulation of the input signal (on the left) derived from applying a spectrotemporal filter to the spectrogram^8,31^. The theta module is modelled by a canonical theta-neuron model^38^, which is fed with the slow amplitude modulation that the model infers from the continuous speech signal. Whenever theta oscillations reach a predefined phase, the model generates a Gaussian pulse, referred to as *theta trigger* (red pulses under ‘Syllable onsets’). Depending on the input, theta triggers appear sooner or later and constitute the model’s estimates of syllable onsets. This information is used to reset gamma activity in the spectrotemporal module (solid arrow from theta to spectrotemporal module). Similarly, the instantaneous frequency/rate of the theta oscillator is used to set the preferred rate of the gamma sequence (dashed red line from theta to spectrotemporal module). Together gamma and syllable units encode the dynamics of the frequency channels in the input. The last (8th) gamma unit represents the model’s estimate about the syllable offset (based on their pre-learned spectral structure); hence it is used to reset syllable units to a common value (upward arrows). During the inference process, the activation level of each syllable unit changes based on bottom-up prediction errors. The identified syllables are readout from the dynamics of syllable units. **b** A simplified diagram of the model indicating the functional connections. The solid arrow from the theta module (*θ*) to gamma units (*γ*) indicates the reset of gamma activity. The dashed red line represents rate information received from the theta oscillation. Finally, the arrow from gamma to syllable units (*ω*), indicates the reset of the syllable units.

An important prerequisite of the model is that it must be able to use the temporal information/cues present in continuous speech, to define syllable boundaries. We hypothesised that internal generative models including temporal predictions should benefit from such cues. To address this hypothesis and to account for recurrent processes occurring during speech recognition^13,32–34^, we used a continuous predictive coding model (described in Methods). Our model explicitly separates *what* from *when*, with *what* referring to the identity of a syllable and its spectral representation (a non-temporal but ordered sequence of spectral vectors), and *when* to the prediction of the timing and duration of syllables as implemented through periodic/oscillatory processes^35,36^. *When* predictions take two forms: syllable *onset* as signalled by a theta module, and syllable *duration* signalled either by exogenous or endogenous theta oscillations that set the duration of a sequence of gamma-timed units (see Methods for details) (Fig. 1).

Precoss retrieves the sensory signal from internal representations about its causes by inverting a generative model. In this case the sensory input corresponds to the slow amplitude modulation of the speech waveform (see Methods section) and a 6-channel auditory spectrogram^37^ of a full natural sentence (bottom rows of Fig. 1), which the model internally generates from the four components depicted in Fig. 1: 1/ a theta oscillation, 2/ a slow amplitude modulation unit in a *theta*-module, 3/ a pool of syllable units (as many syllables as present in the natural input sentence, i.e., from 4 to 25), and 4/ a bank of eight gamma units in a *spectrotemporal*-module. Together, gamma and syllable units generate top-down predictions about the input spectrogram. Each of the eight gamma units represents a phase in the syllable; they activate sequentially and the whole activation sequence repeats itself. Each syllable unit is hence associated with a sequence of eight vectors (one per gamma unit) with 6 components each (one per frequency channel) (see Supplementary Fig. 1). The acoustic spectrogram of a single syllable is generated by the activation of the corresponding syllable unit over the entire syllable duration. While the syllable unit encodes a specific acoustic pattern, the gamma units temporally deploy the corresponding spectral prediction over the syllable duration. Information about syllable duration is provided by the theta oscillation (dashed arrow in Fig. 1), its instantaneous rate affecting the rate/duration of the gamma sequence. Finally, the accumulated evidence about inferred syllable has to be erased before the next syllable is processed. To achieve this, the last (8th) gamma unit (Fig. 1 upwards blue arrows, Supplementary Fig. 1), which encodes the last part of the syllable, resets all syllable units to a common low activation level, enabling new evidence accumulation.

**Fig. 2:**
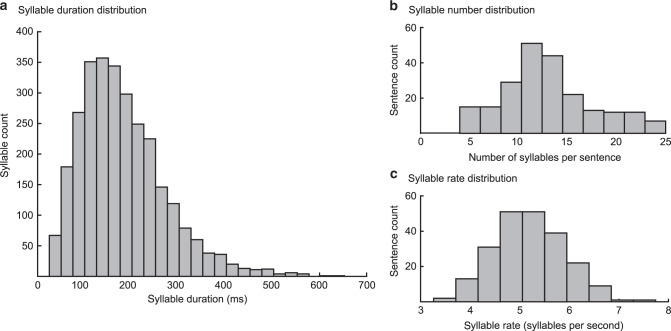
Syllable characteristics. **a** Distribution of the duration of all 2888 syllables in the 220 sentences used for model simulations (mean syllable duration ~182 ms, median ~166 ms). **b** The histogram of the number of syllables per sentence (mean = 13.12; median = 12.5). **c** Distribution of syllable rates across sentences. The mean syllable rate is equal to 5.2 syllables per second (median is equal to 5.15 syllables per second).

The model performance depends on whether the gamma sequence aligns with syllable onsets, and whether its duration is consistent with the syllable duration (50–600 ms, mean = 182 ms in the dataset used here, Fig. 2). To meet these essential criteria, the theta oscillator, as modelled using the Ermentrout-Koppel canonical model^38^, is fed by the dynamics of the slow amplitude modulation of the speech signal inferred from the input, and whenever it reaches a specific, predefined phase, the model generates a Gaussian trigger signalling syllable onset. This setup implies that the operating theta frequency dynamically changes as a function of the inferred slow amplitude fluctuations and can hence estimate syllables duration. This estimation is used to adjust the duration of the gamma sequence (via a hidden variable controlling the rate of the gamma sequence—see Methods section, Eq. (12)).

The model’s estimate about the syllable sequence is provided by the syllable units, which, together with gamma units, generate expected spectrotemporal patterns that are compared with the input spectrogram. The model updates its estimates about the ongoing syllable to minimise the difference between the generated and the actual spectrogram. The activity level increases in those syllable units whose spectrogram is consistent with the sensory input and decreases in the others. In the ideal case, online prediction error minimisation leads to elevated activity in one single syllable unit matching the input syllable.

We quantify the model’s performance by its ability to correctly identify syllables in the input, which is determined by the activity of syllable units within the temporal window between two consecutive activations of the first gamma unit. For each window the model selects the syllable unit that has the highest average activation, the *winning syllable*. Optimal recognition occurs when the winning syllable and the inferred gamma sequence duration correspond to the real identity and duration of the syllable in the input (see corresponding Methods section and Supplementary Fig. 2 for more details).

### Model variants and performance

**Fig. 3:**
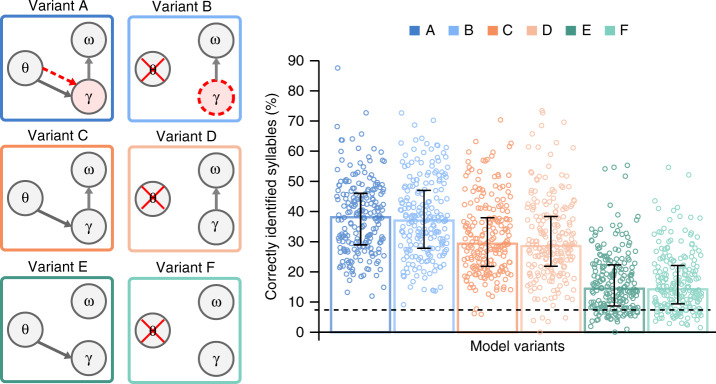
Precoss model variants and their performance. We tested the model’s performance for 6 different architectures (left panel). Columns (dark vs light colours, theta to gamma solid arrow) represent whether the model’s gamma (*γ*) activity is reset by theta (*θ*)—signalled onsets. Rows (differently coloured) correspond to whether syllable units (*ω*) are reset by the last gamma unit (*γ* to *ω* arrow, A, B and C, D) or not reset (E, F). Whenever the γ circle is coloured red, the corresponding model has a preferred rate for the gamma sequence (theta-gamma coupling; A and B). In A, the preferred gamma rate is dynamically set by the theta oscillation (red dashed line from *θ* to *γ* circle), hence it has exogenous theta-gamma nesting. Variant B has internally set preferred gamma rate value (dashed red gamma circle), hence endogenous theta-gamma nesting. The bar plots represent the median performance of each model configuration (*n* = 210 samples). The error bars show 25% and 75% percentiles; the dashed horizontal line indicates the chance level (estimated to be at 6.9%). Pairwise comparisons were performed using the Wilcoxon signed-rank test (2-tailed). *p*-values are given in the Supplementary Table 1. All comparisons except A vs. B, C vs. D and E vs. F are highly significant at a corrected alpha <10^−7^.

The model presented above includes a physiologically motivated theta oscillation that is driven by the slow amplitude modulations of the speech waveform and signals information about syllable onset and duration to a gamma component. This theta-gamma coupling achieves the temporal alignment of internally generated predictions with the syllable boundaries detected from the input (variant A in Fig. 3). To assess the relevance of syllable timing based on the slow amplitude modulation, we compared the performance of model A against that of a variant in which theta activity is not modelled by an oscillation but emerges from the self-repetition of the gamma sequence (Model B, Fig. 3). In this alternative version, the duration of the gamma sequence is no longer controlled exogenously by the theta oscillation, but endogenously using a preferred gamma rate that, as the sequence repeats, results in an intrinsic theta rhythm (Eqs. (12) and (15)). As with the theta oscillation, the duration of the gamma sequence has a preferred rate in the theta range that can potentially adapt to variable syllable durations during the inference process, via prediction errors that change the hidden variable responsible for its rate. With this variant, the model allows for testing a theta rhythm arising from the repetition of the gamma sequence rather than from an explicit stimulus driven oscillator. This scheme where theta emerges from endogenously rhythmic modulations of gamma activity is argued in the literature^39^ as a possible alternative to a separate cortical theta rhythm driving gamma activity^40^. Note that both model versions (A and B) operate with a reset of accumulated evidence by silencing syllable units at the end of each syllable.

In order to more precisely assess the specific effects of theta-gamma coupling and of the reset of accumulated evidence in syllable units, we run additional simulations in two more variants of models A and B. Variants C and D differed by having no preferred gamma rate (Supplementary Fig. 1). Variants E and F further differed from variants C and D by having no reset of accumulated evidence in syllable units (Fig. 3, left panel, Table 1 in Methods). Among all model variants only A has a true theta-gamma coupling, where gamma activity is determined by the theta module (Fig. 3, red dashed arrow), whereas for B the gamma rate is set endogenously (red dashed circle). In all other cases, the rate of gamma activity can freely adapt to the input. To address the impact of the reset of gamma units by exogenous theta triggers we compared models A, C and E, with B, D and F, respectively.

**Table 1:** Triggers for each model configuration.

	Gamma units	Syllable units	Preferred gamma sequence rate
Variant A	T_γ_ = T_θ_	T_ω_ = T_*int*_	f(s) = s_θ_ - s
Variant A’	T_γ_ = Tr no theta	T_ω_ = T_*int*_	f(s) = 1-s
Variant B	no theta	T_ω_ = T_*int*_	f(s) = 1-s
Variant C	T_γ_ = T_θ_	T_ω_ = T_*int*_	f(s) = 0
Variant D	no theta	T_ω_ = T_*int*_	f(s) = 0
Variant E	T_γ_ = T_θ_	T_ω_ = 0	f(s) = 0
Variant F	no theta	T_ω_ = 0	f(s) = 0

Simulations were performed on natural sentences from the TIMIT^41^ speech corpus. Bar plots in Fig. 3 show the median performance for each architecture. Although all model variants performed well above chance, there were significant differences across them (p-values for pairwise comparisons and Bonferroni corrected critical α-value are presented in Supplementary Table 1). Relative to models A and B, performance was significantly lower in models E and F (on average by 23%), and C and D (by 15%) indicating that erasing accumulated evidence about the previous syllable before processing a new syllable is a crucial factor for syllable stream encoding in natural speech.

The comparison of variants A and B versus C and D indicates that theta-gamma coupling, whether stimulus-driven (A) or endogenous (B), significantly improved the model’s performance (on average by 8.6%).

The simulations further show that the model performed best when syllable units were reset after completion of each gamma-units sequence (based on internal information about the spectral syllable structure), and when the gamma rate was driven by theta-gamma coupling irrespective of whether it was stimulus- (red dashed arrow in A) or endogenously-timed (red dashed circle in B). The model’s performance with natural sentences hence depended neither on the precise signalling of syllable onsets via a stimulus-driven theta oscillation, nor on the exact mechanism of the theta-gamma coupling. Although this is a surprising finding, the absence of performance difference between stimulus-driven and endogenous theta-gamma coupling reflects that the duration of the syllables in natural, regular speech is very close to the model’s expectations, in which case there would be no advantage for a theta signal driven directly from the input.

**Fig. 4:**
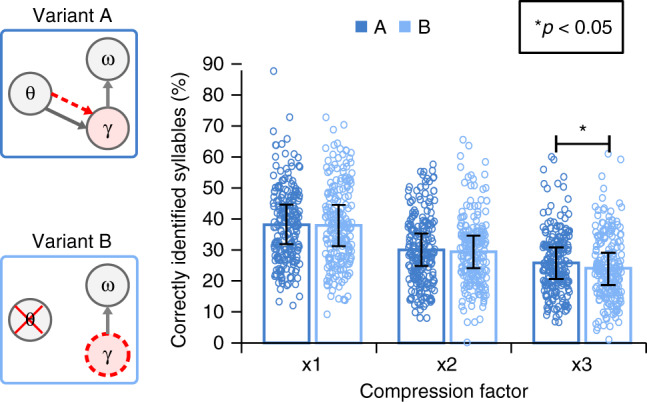
Performance of model variants with and without stimulus-driven theta oscillator on compressed speech. The bar plots represent the mean values (*n* = 210 samples) for each three compression factors (x1 stands for natural speech) with error bars showing standard deviation. For compression factor 3, there is a statistically significant difference in performance between models with stimulus-driven (A dark blue, 25.84% ± 10.2) versus endogenous (B light blue, 24.1% ± 10.6) theta oscillations (1-tailed *t*-test, *p* = 0.0248, *t*-value = 1.97, degrees of freedom = 209, s.d. = 12.76).

To address this point, we run additional simulations on model variants A (theta-gamma coupling through stimulus-driven theta) and B (endogenous theta-gamma coupling via preferred gamma rate) using compressed speech signals (normal speed ×2 and ×3). Behavioural studies^14,42,43^ show that comprehension remains almost intact when speech is compressed by 2 but drops for a compression factor of 3. Under such adverse conditions, stimulus-driven theta-gamma coupling might become beneficial for parsing and decoding syllables. Simulation results with compressed speech stimuli are presented in Fig. 4. For the sake of comparison, we display again performance of variants A and B with natural uncompressed sentences. As expected, overall performance dropped with increased compression factor. However, while for compression factor 2, there was still no significant difference between stimulus-driven and endogenous theta-gamma coupling, a significant difference emerged for compression factor 3, (mean difference = 1.74% [0.28, ∞, 95% CI], Cohen’s *d* = 0.1363, *p* = 0.0248), indicating that a stimulus-driven theta oscillation driving theta-gamma coupling was more advantageous for the syllable encoding process than an endogenously-set theta rate. These simulations therefore suggest that natural speech can be processed with a relatively fixed endogenous theta oscillator but that a more flexible, stimulus-driven, theta oscillator signalling precise syllable timing to the gamma encoding module, might become essential for decoding speech in adverse conditions such as when speech rate is variable.

### Bayesian information criterion

**Fig. 5:**
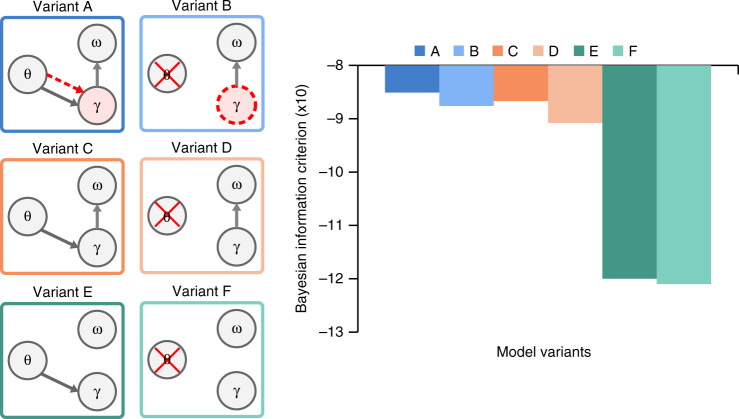
Bayesian information criterion. The left panels represent the Precoss model variants as in previous figures. The right panel represents the BIC value for each model variant. BIC is largest for variant A and smallest for variant F.

The ability of the model to accurately recognise syllables in the input sentence does not take into account the variable complexity of the different models compared. We therefore estimated the Bayesian Information Criterion^44^ (BIC) for each model, which quantifies the trade-off between model accuracy and complexity (Fig. 5).

Variant A, the model in which exogenously driven theta oscillation informs gamma rate and resets the accumulated evidence based on the internal knowledge of syllable structure, showed the highest BIC value. While, the comparison based on syllable recognition accuracy (Fig. 3) could not distinguish between variants A and B, the BIC (Supplementary Table 2) shows that variant A results in more confident syllable recognition than the model without stimulus-driven theta oscillation (Variant B).

Overall, these simulations show that two processes determine the performance of online syllable identification in the model. The first and most important one is the reset of accumulated evidence based on the model’s information about the syllable content (here its spectral structure). The second one is the coupling between theta and gamma periodic processes, which ensures that gamma activity is embedded within a theta cycle corresponding to the expected syllable duration. Importantly, the coupling was more efficient when it arose from a theta oscillator driven by the speech acoustics; the model was marginally more resilient to speech rate variations (Fig. 4), and it had the best accuracy versus complexity trade-off (Fig. 5).

## Discussion

This work focuses on a necessary operation for speech recognition; the parsing of the continuous speech signal into discrete elements. Precoss was exapted from a speech recognition model^26^ inspired by birdsong generation^45^. While most simulations described in Yildiz et al.^26^ were performed on monosyllabic, normalised (in the temporal domain) words (digits), our proposed model extends the approach to natural continuous speech made up of subunits (here syllables) whose order is not known a priori. Since speech is made of linguistic building blocks allowing for (quasi)infinite combinatorial possibilities, this constitutes an important step toward neurobiological realism compared with Yildiz et al.^26^. The model emulates the combinatorial freedom of language by assuming that syllables present in a given sentence can appear in any order. Given real speech statistics, context can reduce—but not generally eliminate—the uncertainty about which syllable might come next. Our main observation suggests that when uncertainty about the identity of the next syllable remains, which is true in most cases, coordination of bottom-up and top-down information flow is critical for recognition and that neural oscillations can enhance this coordination by signalling syllable temporal structure (onsets and duration).

Syllabification is a non-trivial problem that is classically dealt with by off-line methods, e.g., Mermelstein algorithm^46^. It was shown, however, that a theta-range natural oscillator built from reciprocal connections between excitatory and inhibitory leaky integrate-and-fire neurons could achieve highly accurate on-line syllabification^8,31^. The efficiency of such theta oscillator is due to the fact that its intrinsic frequency can adapt, within limits, to that of an external stimulus. In Precoss, we took advantage of the theta oscillation for syllable parsing, using a simplified version of this network including one single canonical theta neuron^38^, whose function is to signal syllable onset based on the slow amplitude modulation of the speech waveform. This simplification was necessary because integrate and fire neurons do not permit to work in a continuous framework. This simplified theta oscillation implementation allowed us to reach around 53% correct syllable onset detection (theta trigger within 50 ms of a real onset), a relatively modest rate, which however was sufficient to demonstrate the advantage of an oscillatory mechanism that can adapt to the acoustics. We indeed compared this model, which includes a stimulus-informed theta oscillation, with a model where theta-gamma coupling is endogenous, i.e., when it arises from the repetition of the gamma sequence at a predetermined rate that flexibly adapts to the variable syllable lengths during the inference process (Eq. (12), Methods). While both models performed similarly for natural speech up to a compression factor of 2, the model with stimulus informed theta oscillator performed better for a compression factor 3. The advantage of the latter model is that gamma activity precisely aligns with the syllables in the input, allowing to encode syllables of variable length as they occur in continuous natural speech.

A critical feature of the model is the coupling between the theta and gamma modules. Many experimental studies indicate that neural theta and gamma activity interact^2,8,47–49^ and most likely that theta organises gamma activity to align neural encoding timing with the temporal structure of the auditory sensory signal. In speech processing, cross-frequency coupling is thought to preserve the hierarchy between phonemes, syllables and even higher order linguistic elements such as phrases^7,50–53^. Here, by having two alternative implementations for the theta rhythm, we effectively modelled two forms of theta-gamma coupling: one in which the gamma sequence is controlled (onset and duration) by the stimulus-driven theta oscillation, and another one in which the theta rhythm emerges from an endogenous gamma sequence. The observation that model variants A and B, which implement one or the other theta-gamma coupling option perform better than the variants without coupling (C, D, E, F), suggests that the nesting of two temporal timescales facilitates on-line syllable recognition, most presumably by aligning top-down and bottom-up information flows with the syllabic structures in the input.

However, we also observed that theta-gamma coupling was not the most important factor for correct performance. Irrespective of theta-gamma coupling the model required explicit reset of syllable units to function at optimal level (Fig. 3 C, D vs. E, F): the high activity level of the winning syllable unit adversely affected the processing of the next syllable, reflecting that syllable units were integrating information across syllable boundaries. This issue was dealt with by incorporating a reset of the accumulated evidence about the previous syllable, which increased performance by up to 15–20%.

Importantly however, model variants A and B, which combine theta-gamma coupling with the reset of accumulated evidence in syllable units, performed better (by 8–10%) than model variants C and D that only relied on syllable units reset, even when the latter had a flexible gamma network rhythm and could in principle have adapted to the variable syllable length in the input sentences.

The performance measure was based on the syllable unit with the highest average activity within a cycle of the gamma network (Supplementary Fig. 2). This measure does not take into account model complexity or the robustness of the model’s syllable estimate. After accounting for both by considering BIC, we found that the model that best accounted for the actual syllables present in the input was variant A, the model in which both the theta rhythm and the gamma network rhythm are stimulus driven. The log-likelihood value used to calculate the BIC value uses the actual values of syllable units together with their precision, and therefore is more sensitive than the performance measure used in Fig. 3. The larger BIC for variant A suggests that even in the cases in which the identity of the syllable unit with highest average activity within a gamma network cycle is the same in both variants, the precision of recognised syllables may be higher for variant A than B. This also suggests that variant A might be more robust to other input modifications such as background noise.

**Fig. 6:**
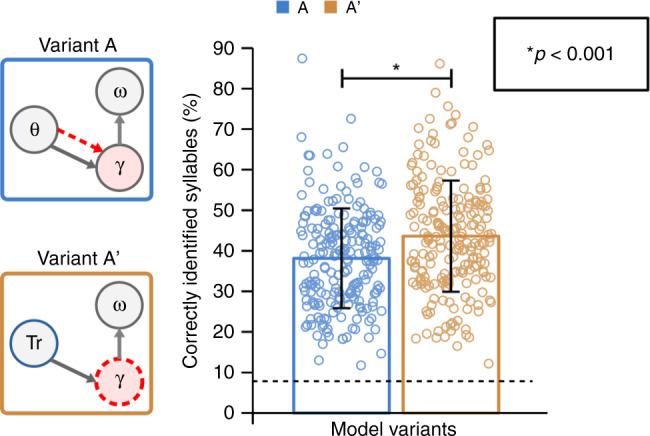
Explicit versus theta signalled onsets. The ideal onset detection condition is modelled by explicitly signalling true syllable onsets (denoted as Tr in blue circle for variant A’). Mean performance value (±s.d.) are shown for each model configuration (*n* = 210 samples). The dashed line on the bar plot corresponds to the chance level 6.9%. Model variant A’ (43.6% ± 13.7), based on variant B (hence features endogenous theta rhythm) with the addition of explicit onset information Tr to reset gamma sequence, performed better than the variant A (38.8% ± 12.13). Pairwise comparison was performed using the 2-tailed paired *t*-test (*t*-value=5.26, *p* = 3.63e-7, degrees of freedom=209, s.d.=15.01).

The absence of a difference in our basic measure of performance between models with stimulus-driven versus endogenous theta-gamma coupling at natural speech rate is a puzzling finding. It suggests that the notion that a theta rhythm drives the gamma network integration window, i.e. that the gamma sequence has a plausible syllable-length duration, is a more important factor than a precise syllable timing. Yet, a possible reason for this observation could be that theta-signalled syllable onsets were too imprecise to provide an optimal gain (as the single neuron theta model only yielded 53% accuracy). To address this point, we ran yet another round of simulations where we compared model performance depending on whether we use the spiking theta oscillator or true syllable onsets to reset the gamma sequence (Fig. 6 model A versus A’).

Performance was higher for variant A’ (Fig. 6, by 5.44% ± 2.04 (95% CI), Cohen’s *d* = 0.363, *p* = 3.63e-7), i.e. when endogenous theta-gamma coupling was driven by true syllable onset information, suggesting that the model can still benefit from accurate syllable onset signalling. Although such an ideal onset detection is non-realistic in a biological system and in natural listening conditions (noise, cocktail-party situations, etc.), this simulation suggests that by improving the way the model handles syllable onset detection the gain provided by exogenous theta-gamma coupling could be further increased. So far, the best performance for on-line syllable boundary detection was obtained using a theta oscillation network of 32 neurons (16 excitatory and 16 inhibitory) bursting together, which made boundary detection more flexible and robust to fluctuations in the speech waveform^8,31^. Increasing the number of theta neurons in the model’s theta module would significantly increase the computational load, yet presumably improve the accuracy of the syllable onset detection.

Although top-down control is constitutively present in the predicting coding implementation, our model lacks the notion that top-down processes involve specific discharge rates, and that, as recently demonstrated, they occur preferentially at a low-beta rate range^12,13,17,21^. The predictive coding model we present here works in a continuous inferential mode, which is discretized only by virtue of syllable timing. In human auditory cortex, bottom-up gamma activity is modulated at the low-beta rate^13,54^, which could offer top-down integration constants that are intermediate between syllables and gamma phonemic-range chunks, and whose advantage could be to smooth the decoding process by providing sequential priors at an intermediate scale between phonemes and syllables. Alternatively, the beta top-down rhythm could be related to expected precision, thus encoding second-order statistics^20^. Expected precision weighs bottom-up prediction errors, hypothesised to work at gamma rate, and could control their impact on the evidence integration process. When the sensory input corresponding to a new syllable arrives, the large prediction error could decrease the estimated confidence in top-down prediction and boost confidence in bottom-up prediction error. If the relative weight of bottom-up and top-down information is carried by low beta activity, we would then expect an alternation between up- and down-going processes at a theta rhythm, a finding that was experimentally observed^13^. An important generalisation for the model would thus consist of adopting a framework that estimates precision^55–57^. These proposals remain speculative, and neurocomputational modelling could be one way to address whether the principle of frequency and temporal division of bottom-up and top-down processing is functionally interesting and whether the low-beta rate for top-down flow is optimal or merely a “just so” phenomenon.

Although our goal was not to design a speech processing model that can compete with those used in the domain of automatic speech recognition^58,59^ it turns out that the notion of neural oscillations can be relevant for the latter^8^, even at the hardware level^60^. Hyafil and Cernak^31^ demonstrated that a biophysically plausible theta oscillator which can syllabify speech *online* in a flexible manner makes a speech recognition system more resilient to noise and to variable speech rates. Here, we confirm that a biologically realistic theta oscillation is useful to signal syllable boundaries, mostly when they are out of natural statistics. More importantly, we show that the coupling between two processing timescales at theta and gamma rates can boost on-line syllable recognition in natural speech. It is hence possible that introducing oscillatory mechanisms in ASR could further improve performance and resilience to noise. For instance, using a basic sampling rate in the low-gamma range as implemented in the current model instead of faster ones as commonly used^61^ could save computing resources without reducing ASR systems performance. The possible coupling of a syllable parsing oscillatory mechanism, with an encoding process that is both exogenously and endogenously timed could also be useful. Finally, using oscillation-based top-down updating, which could deploy predictions about when speech events are expected to happen, a process that most ASR systems do not yet implement^62^, might also turn out to be advantageous.

This theoretical work shows the interest of extending predictive coding approaches to the domain of neural oscillations. This combination permits both to emulate a neurally plausible interface with the real world that is able to deal with the continuous nature of biological stimuli and the difficulty to parse them into elements with discrete representational value, and to provide internal orchestration of the information flow that supply possible deficiencies of interfacing mechanisms. Overall, this study suggests an alternative perspective of combining neural oscillations with predictive coding: namely the longitudinal effect of theta-gamma coupling on the temporal alignment of top-down/bottom-up informational flows during inferential processes.

## Methods

### Speech Input

We used 220 recorded English sentences from the TIMIT database^41^ for our simulations. The sentences were spoken by 22 different speakers (10 sentences each). Overall, those 220 sentences include 2888 syllables. We used the first 10 sentences of the dataset to adjust parameters of the model (Eq. (7) and precisions, Table 2). Thus, these sentences were not included in the model performance analysis.

**Table 2:** Precisions.

	Hidden states	Causal states
Top level	*W*_γ_ = exp(5) *W*_s_ = exp(5) *W*_ω_ = exp(3) *W*_ω_^silent^ = exp(1) *W*_θ_ = exp(7) (if present) *W*_A_ = exp(15) (if present)	*V*_γ_ = exp(1.5) *V*_ω_ = exp(5) *V*_*A*_ = exp(7) (if present)
Bottom level	*W*_f_ = exp(15)	*V*_f_ = exp(10) *V*_A_ = exp(10) (if present)

Input to the model consisted of (1) a time-frequency representation of the sound wave and (2) the slow amplitude modulation of the sound waveform. We used a biologically inspired model of the auditory periphery^37^ to obtain the time-frequency representation, therefore we will refer to it as the auditory spectrogram. The model transforms the auditory signal into 128 logarithmically spaced frequency channels, ranging from 150 Hz up to 7 kHz. We normalised the auditory spectrogram so that the maximum value across all channels is 1 and the minimum 0. After averaging the activity of adjacent channels, we reduced the number of channels to 6, covering the range of frequencies from 150 Hz to 5 kHz. The slow amplitude modulation is calculated following the procedures described in Hyafil et al.^8^ and Hyafil and Cernak^31^. The procedure follows these steps: (1) transform the auditory spectrogram into 32 frequency channels by averaging every 4 channels (from the full 128 channel auditory spectrogram) into 1 channel. (2) convolve with a spectrotemporal filter optimised to signal syllable boundaries. In Hyafil et al.^8^ the output of the spectrotemporal filter, which corresponds to the slow amplitude modulation in the speech waveform, was used to drive a theta oscillator made of integrate and fire model neurons. In Precoss, it is used to drive the theta module that is implemented by a continuous theta neuron.

In summary, each recorded sentence was represented by seven sensory input channels, the slow amplitude modulation (*S*(t)) plus 6 frequency bands (*F*_*f*_(t); *f* = 1, … 6).

### Syllabification

The model’s goal is to recognise syllables on-line, which requires defining syllables in the input to subsequently assess the model’s performance. The TIMIT corpus provides phonemic boundaries labelled by professional phoneticians. This information was passed to Tsylb2, a programme that provides candidate syllable boundaries based on the TIMIT phonemic annotation and on English grammar rules^63^. These boundaries were then used to calculate spectrotemporal patterns ST_*fγω*_ (*f* = 1, … 6, number of frequency channels and *γ* = 1, … 8, number of gamma units) for each syllable *ω* of a given sentence. Firstly, each syllable (*ω*) was divided into 8 equal Δ*T* temporal chunks. Then, syllable spectrotemporal patterns ST_*fγω*_ were calculated by averaging each of the 6 frequency channels (*F*_*f*_(t)) within each of the eight Δ*T* windows (*γ*) for each syllable (*ω*)). We used 6 frequency channels per syllable, thus, the spectrotemporal patterns are matrices with 6 rows and 8 columns for each syllable (*ω*)) (Supplementary Fig. 1).

### Generative model

We used a predictive coding model to parse and recognise individual syllables from the continuous acoustic waveform of spoken English sentences. The core of the predictive coding framework is a hierarchically structured generative model that represents the internal knowledge about the statistics and structure of the external world. During the inference process, the brain inverts the generative model and tries to infer the hidden causes of the sensory input. To invert the generative model, we used the Dynamic Expectation Maximisation algorithm^55,64^, which is based on top-down predictions and bottom-up prediction errors.

We considered a generative model with two hierarchically related levels. At each level *i* in the hierarchy, dynamics are determined by local hidden states (denoted by *x*^*(i)*^) and causal states from the level above (denoted by *υ*^*(i)*^). At the same time, each level generates causal states that pass information to the level below (*υ*^*(i-1)*^). Hidden states are subject to dynamical equations while causal states are defined by static, generally nonlinear transformations of hidden states and causal states. Schematically

Top level (*i* = 2)

1 $${\dot x}^{\left( 2 \right)} = {\it{f}}^{\left( 2 \right)}\left( {{\it{x}}^{\left( 2 \right)}} \right) + {\it{\upvarepsilon }}^{\left( 2 \right)}$$

2 $${\it{v}}^{(1)}{\it{ = g}}^{(2)}\left( {{\it{x}}^{(2)}} \right)+ \upeta^{(2)}$$

The dynamics at this level are only determined by hidden states *x*^*(2)*^. *υ*^*(1)*^ is the output to the level below. The hidden states at this level include hidden states for a theta oscillator, the slow amplitude modulation of speech waveform, syllable units, and gamma units (see below for details). *ε*^*(i)*^ and *η*^*(i)*^ (*i* = 1, 2) stand for random fluctuations for hidden and causal states respectively (the same notation is used in the next sections); their precision determines how predictions errors are weighted^55^. Causal states passing information to the bottom level include causal states for syllable units, gamma units, and the slow amplitude modulation.

Bottom level (*i* = 1)

3 $${\dot x}^{\left( 1 \right)} = {\it{f}}^{\left( 1 \right)}\left( {{\it{x}}^{\left( 1 \right)},{\it{v}}^{\left( 1 \right)}} \right) + {\it{\upvarepsilon }}^{\left( 1 \right)}$$

4 $${\it{v}}^{\left( 0 \right)}{\it{ = g}}^{\left( 1 \right)}\left( {{\it{x}}^{\left( 1 \right)}{\it{,v}}^{\left( 1 \right)}} \right) + \upeta^{\left( 1 \right)}$$

At this level, there are hidden and causal states related to the 6 frequency channels and a causal state for the slow amplitude modulation (which is relayed without modification from the top level).

The output of the bottom level *υ*^*(0)*^ is then compared with the input **Z**(t): a vector containing the slow amplitude modulation and the reduced 6-channel auditory spectrogram.

5 $${\it{v}}^{\left( 0 \right)} = {\bf{{Z}}}\left( {\it{t}} \right)$$

In the following, we write the explicit form of these equations. Supplementary Fig. 3 provides a schematic with all the variables used in the model.

### Top level

The top level has two modules; a theta module with *slow amplitude modulation* and *theta oscillator* units and a spectrotemporal module with *gamma* and *syllable* units.

We have used Ermentrout-Kopell’s canonical model^38^ for the model’s theta oscillation. The theta model receives as an input the slow amplitude modulation signal in the input. Whenever the theta oscillator reaches a predefined phase, the model generates a gaussian trigger, which constitutes the model’s estimate about syllable onsets (called also theta triggers).

The model tracks the slow amplitude modulation in the input with a perfect integrator:

6 $$\frac{{{\it{dA}}}}{{{\it{dt}}}} = 0 + {\it{\upvarepsilon }}_A^{\left( 2 \right)}$$

During inference, *A* generates an equivalent signal that is compared with the slow amplitude fluctuations in the input (see Supplementary Fig. 3); precision weighted prediction errors in generalised coordinates drive Eq. 6 and result in variable *A* tracking the slow amplitude modulations.

We modify the tracked amplitude and use it as an input to the canonical theta model.

7 $$R = 0.25 + 0.21A$$

The first parameter on the right-hand side is chosen so that theta frequency is around 5 Hz whenever *A* is 0. The coefficient for *A* ensures that the range of the resulting rhythm is within the biological theta range (3–8 Hz, tested on the first 10 sentences of the dataset).

The following pair of the equations corresponds to the theta oscillator in the theta module.

8 $$\begin{array}{*{20}{c}} {\frac{{dq_1}}{{dt}} = - kq_2\left( {1 + R + q_1\left( {R - 1} \right)} \right) + \varepsilon _{{\mathrm{q}}_{\mathrm{1}}}^{\left( 2 \right)}} \\ {\frac{{dq_2}}{{dt}} = kq_1\left( {1 + R + q_1\left( {R - 1} \right)} \right) + \varepsilon _{{\mathrm{q}}_{\mathrm{2}}}^{\left( 2 \right)}} \\ {k = \frac{{2\pi \Omega }}{{1000}}} \end{array}$$

Where 1000 is the sampling rate and *Ω* = 5 Hz is the frequency of theta in the absence of input (*A* = 0). The quantity within brackets of the right-hand side of Eq. (8) stands for the normalized instantaneous rate of the theta oscillation (instantaneous rate = *k* ∙ *s*_θ_).

9 $$s_{\uptheta} = 1 + R + q_1\left( {R - 1} \right)$$

The value of *k* ∙ *s*_θ_ determines the interval between two consecutive theta triggers, hence also the interval between two consecutive syllable onsets. As this interval indirectly estimates the syllable duration between signalled onsets, we have used the value of *s*_θ_ to set the preferred rate of the gamma sequence (and by extension gamma sequence duration) as discussed in the next section.

Gamma units are modelled as a stable heteroclinic channel, which results in their sequential activation^65^ (for details see^26,45^). The duration of the gamma sequence depends on the hidden variable *s* (Eq. (12)); which sets the rate of the gamma sequence through *κ*_*2*_ in Eq. (10) and whose dynamics depends on whether the model variant: 1) has no preferred rate for the gamma sequence 2) has a fixed preferred rate or 3) has a preferred rate determined via the exogenous theta oscillation. For the second case, an endogenous rhythm is associated with the gamma sequence (endogenous theta) whose rate is set to generate a rhythm at 5 Hz (Eq. (15)), therefore corresponding approximately to the average syllable duration in English^30^ and to the syllabic rate in our data set (Fig. 2).

In the model, gamma units provide processing windows for the syllable encoding process. The active gamma unit determines which part of the syllable is encoded at each moment of time. For example, if the first gamma unit is active, then the first 1/8 part of the spectral content of a syllable is encoded, if the second gamma unit is active then the second 1/8 part is encoded and so on.

The mathematical equations are adapted from Yildiz et al.^26^.

10 $$\frac{{{\it{dz}}}}{{{\it{dt}}}} = {\it{\upkappa }}_2\left( {\it{s}} \right)\left[ { - \lambda {\mathbf{z}} - {\it{\uprho S}}\left( {\mathbf{z}} \right) + 1} \right] - {\it{\upbeta }}\left( {{\mathbf{z}} - {\mathbf{z}}_0} \right){\it{T}}_\gamma + {\it{\upvarepsilon }}_z^{\left( 2 \right)}$$

11 $$\frac{{{\it{dy}}_{\it{i}}}}{{{\it{dt}}}} = {\it{e}}^{{\it{z}}_{\it{i}}} - {\it{y}}_{\it{i}}\mathop {\sum}\limits_{{\it{j}} = 1}^{{\it{N}}_{\it{\upgamma }}} {{\it{e}}^{{\it{z}}_{\it{j}}}} - {\it{\upbeta }}\left( {{\it{y}}_{\it{i}}{\it{ - y}}_{{\it{i}},0}} \right){\it{T}}_{\it{\upgamma }} + {\it{\upvarepsilon }}_{{\it{y}}_{\it{i}}}^{\left( 2 \right)}$$

12 $$\frac{{ds}}{{dt}} = f\left( s \right) + \varepsilon _{\mathrm{s}}^{\left( 2 \right)}$$

Where

- *i* represents the index of the gamma unit and takes values from 1 to *N*_γ_ = 8.
- **z** is a vector of 8 units encoding the amplitude fluctuations of the gamma units, whereas the vector *y* represents the amplitude of the gamma units scaled to the [0, 1] interval.
- **z**_0_ and **y**_0_ represent the reset values of **z** and **y**, corresponding to the state when the first gamma unit is active (the start of the gamma sequence).
- *T*_γ_ stands for the trigger that gamma units receive from the theta module (Table 1).
- *β*= 0.5 is a scaling factor for theta triggers.
- *S*(**z**) = 1/(1 + e^*-***z**^) is applied component-wise.
- ρ_*ij*_ ≥ 0 is the connectivity matrix, determining the inhibition strength from unit *j* to *i*. Its values are:

13 $${\it{\uprho }}_{{\it{ij}}} = \left\{ \begin{array}{l}0\quad {\it{i}} = {\it{j}}\\ 1.5\quad {\it{j}} = {\it{i}} + 1\\ 0.5\quad {\it{j}} = {\it{i}} - 1\\ 1\quad {\rm{otherwise}}\end{array} \right.$$

The first term on the right-hand side of both Eqs. (10) and (11) is taken from Yildiz et al. (2013)^26^. The trigger term introduced on the right-hand side of Eqs. (10) and (11) ensures that irrespective of the current state of the network, the first gamma unit is activated, and the gamma sequence is deployed from the beginning whenever there is a trigger. When the trigger corresponds to a syllable onset, it ensures that the gamma sequence is properly aligned with the input and therefore that the spectrotemporal predictions temporally align with the input.

We have also modified the equations so that the value of κ_2_ now is a function of the hidden variable *s* (Eq. (12)).

14 $$\begin{array}{l}\kappa _2\left( s \right) = \kappa _0e^{\left( {s - 1} \right)}\\ s\left( {t = 0} \right) = 1\\ \kappa _0 = 0.2625\end{array}$$

The transformation from *s* to *κ*_2_, guarantees that the latter stays positive. *s* was always initialised to *s(t* = 0*)* = 1. This initial value leads to κ_2_ = κ_0_ in Eq. (10), which corresponds to a 25 ms duration for each gamma unit (40 Hz frequency) and an overall duration of 200 ms for the whole gamma sequence. During inference, the value of *s* (and consequently the value of *κ*_2_) can vary depending on the form of *f(s)* in Eq. (12).

15 $$f\left( s \right) = \left\{ {\begin{array}{*{20}{c}} 0 \\ {1 - s} \\ {s_{\uptheta} - s} \end{array}} \right.$$

*f(s)* = 0, corresponds to the no preferred gamma rate condition (no theta-gamma coupling); the value of *s* can change freely. For *f(s) =* 1-*s, s* can change its value (and as a consequence the duration of the gamma sequence) but it will have a tendency to decay back to *s* = 1, that is, the model has a preferred gamma sequence duration of 200 ms; we refer to this as the endogenous theta rhythm and endogenous theta-gamma coupling. Finally, when *f(s) = s*_θ_*-s*, the preferred rate of the gamma sequence is set by the exogenous theta oscillation, corresponding to the exogenous theta-gamma coupling. For all but model variants A, B and A’ *f(s) = 0*, variant A features *f(s) = s*_θ_*-s* (exogenous theta-gamma coupling) and finally, variants B and A’ have endogenous theta-gamma coupling with *f(s) =* 1-*s*. A summary of the model variants can be found in Table 1.

Across the simulations, in the case of the exogenous theta-gamma coupling (variant A, Fig. 3) the average value of κ_2_ across all sentences was equal to 1.23κ_0_ (with standard deviation equal to 0.45κ_0_), which corresponds to a gamma sequence duration around 163 ms. This suggests that the stimulus driven theta oscillation adapts to the syllable duration in the input. Even though the theta frequency was tuned to 5 Hz (corresponding to a gamma sequence duration to 200 ms), the resulting average frequency corresponds to the median syllable duration in our dataset (Fig. 2). In case of variant B (endogenous theta), the gamma rate remained fixed at κ_2_ = κ_0_ (with standard deviation equal to 0.0025κ_0_).

The last module of the top-level contains the syllable units; they represent evidence that the associated syllable corresponds to the syllable in the input. The number of syllable units varies from sentence to sentence and corresponds to the number of syllables in the input sentence plus a silent unit which generates silence; e.g. it should be maximally active whenever there is no signal in the input. The equations for syllable units are:

16 $$\frac{{{\it{d\upomega }}}}{{{\it{dt}}}} = - \left( {{\it{\upomega - \upomega }}_0} \right){\it{T}}_{\it{\upomega }} + {\it{\upvarepsilon }}_{\it{\upomega }}^{\left( 2 \right)}$$

where omega is a vector with as many components as syllables in the sentence (plus silent unit). *T*_ω_ corresponds to triggers (Table 1) that reset the activation level of the syllable units. A trigger drives the activity level of all syllable units towards an equal value *ω*_*0*_. As we will specify below, triggers originated from the last gamma unit, signalling internal expectations about the end of a syllable; in the case of model variants without the reset of syllable units, the trigger was set to 0. Between triggers, syllable units act as evidence accumulators. The activation level of each unit determines its contribution to the generated auditory spectrogram (Eqs. (18) and (21)).

The causal states of the second level pass information to the bottom level:

17 $${\it{\upnu }}_{\it{\upgamma }}^{\left( 1 \right)} = {\mathbf{y}} + {\it{\upeta }}_{\it{\upgamma }}^{\left( 2 \right)}$$

18 $${\it{\upnu }}_{\it{\upomega }}^{\left( 1 \right)} = \frac{{{\it{e}}^{{\it{ - \upomega }}}}}{{{\sum} {{\it{e}}^{{\it{ - \upomega }}}} }} + {\it{\upeta }}_{\it{\upomega }}^{\left( 2 \right)}$$

19 $${\it{\upnu }}_A^{\left( 1 \right)} = {\it{A}} + {\it{\upeta }}_A^{\left( 2 \right)}$$

Equation (17) corresponds to the 8 scaled gamma units (Eq. (11)); that have sequential activation. Equation (18) corresponds to the syllable units; where we used the softmax function to scale the activity of the syllable units. Since all the syllables in the input are present in the syllable pool, prediction error (the difference between predicted and actual spectrotemporal patterns at the first level) will be minimised when the causal state of the corresponding syllable unit in the model is driven close to 1 while all others are driven close to 0. Finally, Eq. (19) sends information about the current estimate of the slow amplitude modulation.

### Bottom level

The bottom level contains variables related to the amplitude fluctuations of the frequency channels as well as the slow amplitude modulation.

The amplitude fluctuations of the frequency channels are modelled with a Hopfield attractor-based neural network^66^. The following equations were adapted from Yildiz et al.^26^

20 $$\frac{{{\it{d}}{\mathbf{x}}^{\left( 1 \right)}}}{{{\it{dt}}}} = {\it{\upkappa }}_1\left[ { - {\it{D}}{\mathbf{x}}^{\left( 1 \right)} + {\it{W}}{\mathbf{{tanh}}}\left( {{\mathbf{x}}^{\left( 1 \right)}} \right) + {\it{I}}} \right] + {\it{\upvarepsilon }}^{\left( 1 \right)}$$

21 $${\it{I}}_{\it{f}} = \mathop {\sum}\limits_{{\it{\upgamma }} = 1}^8 {\mathop {\sum}\limits_{{\it{\upomega }} = 1}^{{\it{N}}_{{\it{syl}}}} {{\it{\upupsilon }}_{\it{\upgamma }}^{\left( 1 \right)}{\it{\upupsilon }}_{\it{\upomega }}^{\left( 1 \right)}{\it{P}}_{{\it{f\upgamma \upomega }}}} }$$

**x**^**(1)**^ is a vector with 6 components (one per frequency channel), *D* is a diagonal self-connectivity matrix and *W* is an asymmetric synaptic connectivity matrix; they were designed so that the Hopfield network has a global attractor whose location depends on vector **I**^26^. In Eq. (21), **ν**_**γ**_^(1)^ and **ν**_**ω**_^(1)^ are the causal states for the gamma and syllable units from the top level (Eqs. (17) and (18)). P_fγω_ is defined from the spectrotemporal patterns ST_fγω_ associated with each syllable as follows:

22 $${\it{P}}_{{\it{f\upgamma \upomega }}} = \mathop {\sum}\limits_{{\it{i}} = 1}^6 {{\it{D}}_{{\it{fi}}}{\it{ST}}_{{\it{i\upgamma \upomega }}}} - \mathop {\sum}\limits_{{\it{i}} = 1}^6 {{\it{W}}_{{\it{fi}}}{\mathrm{tanh}}\left( {{\it{ST}}_{{\it{i\upgamma \upomega }}}} \right)}$$

Because the vector *I*_*f*_ determines the global attractor, sequential activation of the gamma units makes the global attractor change continuously over time and generate the pattern corresponding to syllable ‘ω’ when *υ*^*(1)*^_ω_ *= 1* and *υ*^*(1)*^_not ω_ *= 0*.

The outputs of this level are the states of the Hopfield network, which predict the activity of the frequency channels in the input, and the causal state associated with the slow amplitude modulation (relayed from the top level):

23 $$\begin{array}{c}{\it{\upupsilon }}_{\it{f}}^{\left( 0 \right)} = {\mathbf{x}}^{\left( 1 \right)} + {\it{\upeta }}_{\it{f}}^{\left( 1 \right)}\\ {\it{\upupsilon }}_A^{\left( 0 \right)} = {\it{\upupsilon }}_A^{\left( 1 \right)} + {\it{\upeta }}_A^{\left( 1 \right)}\end{array}$$

These quantities are compared with the slow amplitude modulation (*S(t)*) and frequency channels (F_*f*_(t)) in the input signal:

24 $$\begin{array}{l}{\it{\upnu }}_A^{\left( 0 \right)} = {\it{S}}\left( {\it{t}} \right)\\ {\it{\upnu }}_{\it{f}}^{\left( 0 \right)} = {\it{F}}_{\it{f}}\left( {\it{t}} \right)\end{array}$$

The discrepancy between top-down predictions and sensory input is propagated back in the hierarchy to update hidden state and causal state estimates so that prediction errors at every level of the hierarchy are minimized.

The values of all parameters used in the model, as well as precisions for hidden and causal states for both levels, are presented in Tables 3 and 2 respectively.

**Table 3:** Parameter values.

	Hidden states
Top level	*Ω* = 5
	*λ* = 0.125
	*β* = 0.5
	*σ* = 0.15
Bottom level	*κ*_1_ = 2

### Resets/triggers and model variants

To ensure that predictions are temporally aligned with the input, the model needs to align the gamma network with syllable onsets. Moreover, ideally, evidence accumulation should be reset before syllable onset. Although both resets could in principle be driven by prediction errors, our basic model also involves explicit resets.

When present, the trigger to reset gamma units (denoted by *T*_γ_ in Eqs. (10) and (11)) was driven either by exogenous theta signalled onset triggers (referred to as theta triggers *T*_θ_) or by explicitly provided onsets *Tr*.

Theta-triggers *T*_θ_: A Gaussian pulse was generated whenever the phase of the exogenous theta oscillation reached a specific phase:

25 $$\begin{array}{l}{\it{T}}_\theta = {\it{e}}^{{\it{ - }}\frac{{\left( {{\it{q}}_{1{\it{n}}}{\it{ + }}1} \right)^2{\it{ + q}}_{2{\it{n}}}^2}}{{2{\it{\upsigma }}^2}}}{\kern 1pt} \;\;\;\;\;\;\\ {\it{q}}_{1n}{\it{ = }}\frac{{{\it{q}}_1}}{{\sqrt {{\it{q}}_1^2{\it{ + q}}_2^2} }}{\it{,}}\quad {\it{q}}_{2n}{\it{ = }}\frac{{{\it{q}}_2}}{{\sqrt {{\it{q}}_1^2{\it{ + q}}_2^2} }}\end{array}$$

When present, the reset to syllable units (*T*_ω_, Eq. (16)) was driven by the model’s knowledge about syllable spectral structure (e.g. knowledge that each syllable is a sequence of 8 spectral target points). Since the last (eighth) gamma unit signals the end of the syllable, it can define a trigger that we refer as the internal trigger (denoted as *T*_int_):

26 $${\it{T}}_{int} = {\it{y}}_8$$

In summary, to reset gamma units the model uses theta triggers, which are derived from the slow amplitude modulation of the sound waveform, whereas to reset syllable units the model uses internal information about the spectral structure of syllables - *T*_int_. To explore the relative importance of each resetting mechanism for the overall performance of the model, we compared different model variants (Table 1).

### Model output

To define the syllables identified by the model, we considered the time average of the causal state (*v*_ω_, Eq. (18)) of each of the syllable units taken within the boundaries defined by the gamma sequence (Supplementary Fig. 2). We have calculated for how long the unit with highest average activation between the internally signalled boundaries corresponds to the syllable in the input at that time. Thus, for each sentence, we calculate the percentage of the total sentence duration which was correctly identified by the model (Supplementary Fig. 2).

### Statistical analyses

As described in the previous section, a single number (% correctly identified sentence duration) describes the performance of the model for each sentence. Simulations were run on 210 sentences, and the performance of each model architecture is thus described by a vector of 210 numbers. The non-parametric Wilcoxon signed-rank test for repeated measures was used to compare models’ performance. The Bonferroni correction was used to control for multiple comparisons. Supplementary Table 1 reports p-values for pairwise comparisons for the model variants presented in Fig. 3. Table also includes the adjusted α-value.

For the simulations with compressed speech, we only compared the performance difference between 2 model variants for each condition separately. Each pair was compared using the paired t-test (we only compare performance difference of the 2 model variants (A and B) for a particular compression factor).

For the simulations presented in Figs. 4 and 6, Cohen’s d value was calculated as the difference between the means of each group, divided by the standard deviation calculated from differences in observations.

Finally, to estimate the chance level, the following steps were performed: 1) for each sentence in the database, we mimicked the sequence of model-selected syllables (Supplementary Fig. 2, panel **c**) by randomly choosing 2) a duration of “selected” syllable from the syllable duration distribution in Figs. 2a and 3) an identity from the syllables of the corresponding sentence. Thus, we have calculated what would be the model’s performance when syllable identity and duration were selected by chance. For each sentence, the procedure was repeated 1000 times and the mean performance value was stored (a chance level for a sentence). Furthermore, the whole procedure was repeated 1000 times on all 220 sentences in the database, and the median value of the resulting distribution was selected as a chance level for the whole dataset, which was equal to 6.9%.

### Model comparison

We have calculated the Bayesian information criterion value based on the posterior probability of the true syllable sequence **s** (Supplementary Fig. 2d) for each sentence and model variant m; *p(****s*** | m*)*. This quantity also represents the likelihood of the model given the input (true syllables that the model aims to recover).

Similar to the syllable recognition accuracy presented in Fig. 3, the likelihood value is based on the dynamics of the second level causal states (Supplementary Fig. 2) but it also takes into account the precisions (confidence) of the estimated dynamics of each variable (e.g. dynamics of syllable and gamma units in Supplementary Fig. 2a, b).

To derive the BIC, we first calculated model m likelihood for each sentence *i*, and each moment of time (for convenience we do not write the explicit time dependence of *D* and **ν**_μ_ on the right-hand side of the equations below):

27 $$\begin{array}{l}p\left( {{\mathbf{s}}_i\left( t \right)|m} \right) = {\int} {dz\,p\left( {{\mathbf{s}}_i,{\mathbf{z}}|m_i} \right)} \equiv \frac{{\sqrt {\det \,D} }}{{\sqrt {(2\pi )^d} }}{\int} {dz\,\exp \left[ { - \frac{1}{2}\left( {\left( {{\mathbf{\upsilon }} - {\mathbf{\upsilon }}_\mu } \right)^TD\left( {{\mathbf{\upsilon }} - {\mathbf{\upsilon }}_\mu } \right)} \right)} \right]} \\ D \equiv \left[ {\begin{array}{*{20}{c}} A & C \\ {C^T} & B \end{array}} \right],\,\upsilon \equiv \left[ {\begin{array}{*{20}{c}} {\mathbf{s}} \\ {\mathbf{z}} \end{array}} \right]\end{array}$$

**s**_*i*_ is the vector of syllable units as they appear in the input sentence *i* at time *t* (Supplementary Fig. 2d). *D* is the overall conditional precision matrix, which we separate into the components corresponding to the syllable units *A*, components corresponding to the other causal states *B*, and their interaction terms *C*. **ν** represents the vector of causal states in the second level and **ν**_μ_ the model’s estimated means. It contains the syllable units **s** (mean values **s**_μ_, Supplementary Fig. 2a) and all other causal states represented here by **z** (mean values **z**_μ_) for variants B, D, F **z** represents the dynamics of the gamma units, e.g. Supplementary Fig. 2b and, for variants A, D and E, the gamma units and the slow amplitude modulation.

After integration over **z**, the likelihood of the model given the true syllables is:

28 $$p\left( {{\mathbf{s}}_i\left( t \right)|m_i} \right) = \frac{{\sqrt {\det \,D} }}{{\sqrt {\left( {2\pi } \right)^{n_s}} \sqrt {\det B} }}\exp \left[ { - \frac{1}{2}\left( {{\mathbf{s}}_i - {\mathbf{s}}_\mu } \right)^T\left( {A - CB^{ - 1}C^T} \right)\left( {{\mathbf{s}}_i - {\mathbf{s}}_\mu } \right)} \right]$$

This value was calculated for each moment of time, thus, to get the value for the whole sentence we calculated the average value of log(*p(****s***_***i***_*(t)*|m*)*) per syllable and sum across all syllables in the sentence.

29 $$\log p\left( {{\mathbf{s}}_i|m} \right) \approx \mathop {\sum}\limits_{j = 1}^{N_i} {\mathop {\sum}\limits_k {\Delta t\,\frac{{\log \left( {p\left( {{\mathbf{s}}_i\left( {t_k} \right)|m} \right)} \right.}}{{T_j}}} }$$

Where *T*_*j*_ is the duration of syllable *j* in the sentence *i*, *N*_*i*_ is the number of syllables in the sentence and Δ*t* = 1 ms.

Equations (28) and (29) represent the approximate log-likelihood value for model m given the sentence *i*. Thus, the value for the whole dataset is:

30 $$\log p\left( {{\mathbf{s}}|m} \right) = \mathop {\sum}\limits_i {\log p\left( {{\mathbf{s}}_i|m_i} \right)}$$

This was used to approximate the Bayesian information criterion (BIC), which considers also the number of free variables in the model.

31 $$BIC\left( m \right) = \log p\left( {{\mathbf{s}}|m} \right) - \frac{{N_p\left( m \right)}}{2}\log N_{{\mathrm{sentences}}}$$

Where *N*_*p*_*(*m*)* is the number of free variables for model variant m, which includes state precision values (Table 2), number of resets and the presence of gamma rate information. Thus, the most complex variant A would have 14 (precisions) + 2 (reset of gamma units by theta signalled onsets, reset of syllable units by the last gamma unit) + 1 (rate) = 17 parameters. On the other hand, the simplest version F would have only 10 parameters, no precisions values for theta and slow amplitude modulations (−(1 + 3)), values for resets of syllable and gamma units (−2) and no preferred gamma rate value (−1). The number of parameters for each model variant is given in Table 4.

**Table 4:** Number of free parameters for each model configuration.

	Number of parameters
Variant A	17
Variant B	12
Variant C	16
Variant D	11
Variant E	15
Variant F	10

### Reporting summary

Further information on research design is available in the Nature Research Reporting Summary linked to this article.

## Supplementary information

Supplementary Information

Peer Review File

Reporting Summary

## Data Availability

Source data files are provided for Figs. 3, 4 and 6. Subset of the TIMIT dataset^41^ used for simulations and the corresponding Tsylb2^63^ generated syllable boundaries can be found at: https://github.com/sevadah/precoss/tree/master/data_construction. Source data are provided with this paper.

## Source data

Source Data

## Code availability

Simulations were performed with DEM Toolbox in SPM^64^ using MATLAB 2018b, The MathWorks, Inc., Natick, Massachusetts, United States. Custom code is available at: https://github.com/sevadah/precoss. Source data are provided with this paper.
