# Supplementary material for: Combining predictive coding and neural oscillations enables online syllable recognition in natural speech
Source: Nat Commun. 2020 Jun 19;11:3117. doi: 10.1038/s41467-020-16956-5 (PMC7305192; doi:10.1038/s41467-020-16956-5)
Supplement: Supplementary file 1 — Supplementary Information [file 41467_2020_16956_MOESM1_ESM.pdf]

Reviewers' Comments:

Reviewer #1:
Remarks to the Author:
The authors present a model that combines a predictive coding approach with an existing model of an oscillator-based system. I think this word makes a solid and worthwhile contribution to the literature, and I commend the authors for the clarity with which their work was presented. However, the impact of the work could be strengthened by showing same result on non-normalized data, testing the model against existing neural data, generating predictions through simulation, and/or testing against non-oscillating ASR models. The case for predictive coding and oscillations is not a sweeping theoretical novelty, but is nonetheless an important line to pursue if the above strengthening points can be added. That said, my major concern is that the authors normalize their speech data without justification, and that they make claims that just are not supported by what they show.
I see a few problems that need to be addressed. First, the approach, while a step forward from the authors' previous models, still completely ignores the actual behavioral goals of speaking, which is, at the least, communicating meaning (viz., words, phrases, sentences, linguistic structure). Unless the authors want to claim that they think that syllable detection is somehow orthogonal or independent from word recognition or prosodic processing, they need to dial back their claims about speech and anything higher than then syllable. They are making a model of syllable onset detection, not speech processing or sentence processing. The authors need to dial back some of the claims they are making regarding this issue. All references to "speech" and "sentence processing" should be changed to "syllable" and "syllable processing," respectively, including, importantly, the title. Speech processing is much more than syllable detection and prediction of the timing of normalized syllables (such that they are forced to be the same length) – what is shown in this paper – has nothing little to do with perceiving words, never mind sentence processing. For example, what evidence is there that the cochlea (or even auditory cortex) normalizes the length of syllables? The authors say several time in the paper that biological oscillators are more flexible and can deal with the non-stationarity and aperiodicity of speech, but then why do they normalize their training data? Why is predictive coding only about syllable length/ onsets? Lastly on this point, why focus on syllable duration or syllable onset as what is being predictively coded? Do we really know how long a syllable lasts? Does having syllables of unexpected lengths really cause speech intelligibility to break? If so, how come I can understand "happy" and "haaaaappy" as the same word? Without a convincing motivation, normalizing all the data to be the same size seems like stacking the cards firmly in one's favor (lines 161-171) and has no biological plausibility.
Second, the way the authors use the term "optimization" is imprecise – optimal compared to what? They are only comparing variants of their model. They should compare to previous instantiations of their model, an ASR model, and test against real neural data if they really want to use the term 'optimal'. Another way to ameliorate this issue would be to do some simulations and predict either oscillatory patterns in neural data or predict model performance on unseen TIMIT data, for example, by testing on untrained data from a larger variety of speakers.
Finally, in the end, the model still only tracks the syllable envelope, is that what that authors think speech is for? In other words, the point of listening to natural sentences is not to accurately predict syllable durations. So the case for why what this model captures is crucial needs to be more strongly made.
Minor points
- Citation 9 isn't quite accurate, that paper doesn't deal with "what is going to be said next" in a linguistically sophisticated way at all. A reference that deals with actual linguistic structure would be more appropriate.
- Change Figure 1. Caption to replace "sentence processing" with "syllable processing" and remove the word 'natural' – syllables were normalized afterall
- Hierarchical encoding of phonemes with syllables is conjecture – I am aware that this is the main

tenet of the Giraud & Poeppel paper, but where is the evidence that phonemes are encoded and not emergent? Recent ecog work from the Chang group suggests that phonetic features might reorganize to form syllables and this might mean that features are what gamma is encoding (even though I am against functional interpretation of bands)

Reviewer #2:
Remarks to the Author:
This study reports on a computational speech recognition model that combines a theta-gamma oscillatory achitecture (used and established before in a bottom-up manner by the same group; Hyafil et al., eLife 2015) with a predictive-coding-based model arichtecture.
The larger framework here is provided by the senior author's model of nested theta--gamma oscillations being involved in the neural speech comprehension processes. This is an interesting extension of the extant model, borrowing heavily from a computational model by the Kiebel group (Yildiz et al.) on bird song.

As to be expected from such a paper, a family of model variants are compared in their performance (ie % syllables correctly identifed). The final results (Fig. 3) are compelling.
However, the manuscript seems not to provide some of the critical comparisons or model variants that would make this a compelling new model (see below for more detailed comments on this).

Put more generally, while I might have missed some subtleties of the computational models and the merit these might have for experts who have implemented and used the Hyafil and Yildiz models themselves, I did not walk away from this manuscript convinced that a truly new architecture has been shown here, or that we we think should think differently about speech recognition and speech representation now.

Further comments:
-It was a bit disappointing, if understandable, that the units in the model were tuned to very rigid/unnatural rates (ie the theta module operated on strictly 5 Hz throughout; there were always precisely 8 gamma units etc.). Seeing how the model deals with precisely the kind of variations that occurs in natural speech (as the authors have tried with the varying syllable SOAs, to be fair) might have been more informative for future application of the model in cogniitve neuroscience/automatic speech recognition.

-Related, it remained puzzling to me why such a submission would operate only/report only on 30 sentences. I am not asking for a closer link to neural data or human speech comprehension data per se (although this would have also made for a stronger paper, most likely). But the breadth and depth of the speech materials these models are tested on should be increased.

-Most importantly, how can we generalise from these results that a theta module is the critical one, if no differently-tuned module was tested (eg varying the low rate between 1 and 10 Hz)? Again, a richer set of test cases would have made for a more trustworthy claim here. (cf. line 274: "For the sake of parsimony, this was not implemented in the present work.")

-At first glance more of a detail: why the very conservative false discovery rate threshold of q = 0.001? Figure 3 and Table S2 give the impression that the model variants differed quite profoundly from each other, but this also raises suspicion – why so vast differences between models, and would this have been different with more/more diverse speech materials as test cases?

Reviewer 1

*The authors present a model that combines a predictive coding approach with an existing model of an oscillator-based system. I think this word makes a solid and worthwhile contribution to the literature, and I commend the authors for the clarity with which their work was presented. However, the impact of the work could be strengthened by showing same result on non-normalized data, testing the model against existing neural data, generating predictions through simulation, and/or testing against non-oscillating ASR models. The case for predictive coding and oscillations is not a sweeping theoretical novelty, but is nonetheless an important line to pursue if the above strengthening points can be added. That said, my major concern is (**i**) that the authors normalize their speech data without justification, and that they make claims that just are not supported by what they show.*

1. *I see a few problems that need to be addressed.*

(i) *First, the approach, while a step forward from the authors' previous models, still completely ignores the actual behavioral goals of speaking, which is, at the least, communicating meaning (viz., words, phrases, sentences, linguistic structure). Unless the authors want to claim that they think that syllable detection is somehow orthogonal or independent from word recognition or prosodic processing, they need to dial back their claims about speech and anything higher than then syllable. They are making a model of syllable onset detection, not speech processing or sentence processing. The authors need to dial back some of the claims they are making regarding this issue. All references to "speech" and "sentence processing" should be changed to "syllable" and "syllable processing," respectively, including, importantly, the title. Speech processing is much more than syllable detection and prediction of the timing of normalized syllables (such that they are forced to be the same length) – what is shown in this paper – has nothing little to do with perceiving words, never mind sentence processing.*

(ii) *For example, what evidence is there that the cochlea (or even auditory cortex) normalizes the length of syllables? The authors say several time in the paper that biological oscillators are more flexible and can deal with the non-stationarity and aperiodicity of speech, but then why do they normalize their training data?*

(iii) *Why is predictive coding only about syllable length/onsets? Lastly on this point, why focus on syllable duration or syllable onset as what is being predictively coded? Do we really know how long a syllable lasts? Does having syllables of unexpected lengths really cause speech intelligibility to break? If so, how come I can understand "happy" and "haaaaappy" as the same word? Without a convincing motivation, normalizing all the data to be the same size seems like stacking the cards firmly in one's favor (lines 161-171) and has no biological plausibility.*

We thank Reviewer 1 for bringing up these important points about the model's goal and performance. We admit that several statements that we made were more ambiguous than intended, and we adjusted the text to clarify them.

(i) We fully acknowledge that using sentences with normalized syllables was a weakness of the model and we have addressed the issue in the revised version of the model.

Although we never claimed the auditory system does any sort of syllable normalization, we had several technical reasons for doing so in the previous version of the model. They were mostly related to the methodology used to create the model's "gamma sequence", and to the stored information about spectrotemporal patterns of each syllable. Syllable normalization allowed us to have a streamlined representation of each syllable in the model's memory.

Following the reviewers' comments (the same concern about using normalized syllables was raised by Reviewer 2 as well), we have modified several components of the model, which now enables us to use natural sentences (with natural syllable duration) for simulations:

- **The theta module is now based on the canonical theta neuron model by G.B. Ermentrout and N. Kopell [1].**
- **Instead of speech envelope, we use the slow amplitude modulation of the speech soundwave calculated as in Hyafil et al. [2] (output of a spectrotemporal filter specifically trained to signal syllable onsets).**
- **The duration of the gamma sequence, as well as the frequency of theta oscillations, are no longer fixed. The model can adapt the gamma sequence duration on the basis of prediction errors, and the exact theta frequency now depends on the input stimulus through the slow amplitude modulation.**

Detailed descriptions of the modifications are included in the updated Methods section.

(ii) We can only agree with the reviewer that speech perception is much more than just recognizing syllables. However, "on-line" syllable identification within natural sentences (what the model does) is a key step towards that goal. The model implements dual-scale decoding of sentences that structurally incorporates the notion of endogenous syllable representations and top-down control, a notion that is absent in most models including ASR algorithms[3].

(iii) Predictive coding was used to predict the spectrotemporal decompositions of the sound waveform, but not explicitly syllable duration. Surely, the model had intrinsic information about syllable duration (associated with the duration of the gamma sequence) and it attempted to extract/filter syllable onsets from possible cues on the envelope, but those two functions were mostly unrelated to the predictive aspect of the model. The model uses predictive coding to derive the dynamic of the input envelope and to change the activity level of the syllable units in the process of syllable identification. Syllable units changed their activation level based on bottom-up prediction errors and their activation level determined the model's prediction about the spectrotemporal pattern in the input at each moment. As a result, the model identifies individual syllables online from the continuous sentence. Furthermore, we now include a model configuration with no internal syllable duration information, in which the model only "knows" the sequential spectral patterns of syllables (in this case represented by the 8 spectral vectors in the spectral space - one per gamma unit). In this degraded variant of the model "happy" and "haaaappy" would be undistinguishable.

2. *Second, the way the authors use the term "optimization" is imprecise – optimal compared to what? They are only comparing variants of their model. They should compare to previous instantiations of their model, an ASR model, and test against*

real neural data if they really want to use the term 'optimal'. Another way to ameliorate this issue would be to do some simulations and predict either oscillatory patterns in neural data or predict model performance on unseen TIMIT data, for example, by testing on untrained data from a larger variety of speakers.

We agree that the use for the term 'optimal' was somewhat improper as our goal was not to develop an optimal system that could challenge current ASR systems, but to explore how the brain could possibly make use of different information encoding principles, that is, neural oscillations for information packaging and predictive coding for the continuous dynamic interaction of bottom-up and top-down information flows.
We have thus removed the term "optimization" from the title, and we use it cautiously in the manuscript. We now also compare more model variants (with implicit and explicit theta oscillations, and a model without any explicit oscillatory activity). Finally, we have increased the number of sentences used in model simulations (220 sentences instead of 30).

3. *Finally, in the end, the model still only tracks the syllable envelope, is that what that authors think speech is for? In other words, the point of listening to natural sentences is not to accurately predict syllable durations. So, the case for why what this model captures is crucial needs to be more strongly made.*

We hope that it is now clearer from the text that the model does not only track the syllable envelope, but "*identifies"* syllables "*in an on-line"* manner using predictive coding, that is, it tracks and predicts both detailed spectrotemporal content *and* changes in the envelope. What we argue is that temporal predictions about syllable duration are necessary to predict/derive the expected spectrotemporal component of the syllables. However, it is the spectrotemporal component that the model uses to identify the correct syllable. Furthermore, as we indicate in response to the previous point, we have also added model configurations without an implicit theta rhythm, hence without internal expectations about syllable duration. Those model configurations only "know" the spectral structure of syllables as a sequence of 8 spectral points that form a syllable, without any expectations about their overall duration.

4. *Minor points*

(i) *Citation 9 isn't quite accurate, that paper doesn't deal with "what is going to be said next" in a linguistically sophisticated way at all. A reference that deals with actual linguistic structure would be more appropriate.*
(ii) *Change Figure 1. Caption to replace "sentence processing" with "syllable processing" and remove the word 'natural' – syllables were normalized afterall*
(iii) *Hierarchical encoding of phonemes with syllables is conjecture – I am aware that this is the main tenet of the Giraud & Poeppel paper, but where is the evidence that phonemes are encoded and not emergent? Recent ecog work from the Chang group suggests that phonetic features might reorganize to form syllables and this might mean that features are what gamma is encoding (even though I am against functional interpretation of bands).*

(i) We thank the reviewer for the suggestions; we have updated citation 9, although the intention was to indicate that the speech perception process can be split into two

information components: what (syllable identity) was the signal and when (e.g. syllable onset information by theta/readout window for a syllable identification) occurred.
(ii) We have updated the title of the manuscript and did the corresponding changes in the manuscript text and figure captions.
(iii) Finally, we must clarify that we did not claim that gamma in the model encodes phonemes. Even though timescales of phonemes in natural speech overlap with the typical range of gamma cortical oscillations, in our model there is no precise correspondence between gamma units and phonemes. The model explores if there is an advantage of having *gamma range* encoding within syllable boundaries, by considering theta-gamma nesting.

Reviewer 2

This study reports on a computational speech recognition model that combines a theta-gamma oscillatory architecture (used and established before in a bottom-up manner by the same group; Hyafil et al., eLife 2015) with a predictive-coding-based model architecture. The larger framework here is provided by the senior author's model of nested theta--gamma oscillations being involved in the neural speech comprehension processes. This is an interesting extension of the extant model, borrowing heavily from a computational model by the Kiebel group (Yildiz et al.) on bird song.

As to be expected from such a paper, a family of model variants are compared in their performance (ie % syllables correctly identified). The final results (Fig. 3) are compelling. However, the manuscript seems not to provide some of the critical comparisons or model variants that would make this a compelling new model (see below for more detailed comments on this).

Put more generally, while I might have missed some subtleties of the computational models and the merit these might have for experts who have implemented and used the Hyafil and Yildiz models themselves, I did not walk away from this manuscript convinced that a truly new architecture has been shown here, or that we think should think differently about speech recognition and speech representation now.

Further comments:
5. -It was a bit disappointing, if understandable, that the units in the model were tuned to very rigid/unnatural rates (ie the theta module operated on strictly 5 Hz throughout; there were always precisely 8 gamma units etc.). Seeing how the model deals with precisely the kind of variations that occurs in natural speech (as the authors have tried with the varying syllable SOAs, to be fair) might have been more informative for future application of the model in cognitive neuroscience/automatic speech recognition.

We thank Reviewer 2 for bringing up these issues. The revised version of the model now uses natural sentences with non-normalized syllables with natural duration. Furthermore, the frequency of the "theta" oscillation in the different model variants is now stimulus-driven and not rigidly fixed to 5Hz, which is the operating frequency during rest (when there is no signal). Finally, even though we still have 8 gamma units per syllable, the duration of each unit is not fixed and can dynamically change either based on prediction errors or informed by the theta module.

6. -Related, it remained puzzling to me why such a submission would operate only/report only on 30 sentences. I am not asking for a closer link to neural data or human speech comprehension data per se (although this would have also made for a stronger paper, most

likely). But the breadth and depth of the speech materials these models are tested on should be increased.

We thank the reviewer for prompting us to increase the speech material. We now present results from simulations on 220 sentences (all sentences in training data-set of TIMIT corresponding to one dialect). These sentences contain on average around 13 syllables (more details on Figure 2 in the Manuscript).

7. -Most importantly, how can we generalise from these results that a theta module is the critical one, if no differently-tuned module was tested (eg varying the low rate between 1 and 10 Hz)? Again, a richer set of test cases would have made for a more trustworthy claim here. (cf. line 274: "For the sake of parsimony, this was not implemented in the present work.")

Although we did not include the following results in the revised manuscript, we have tested the model performance when the theta rhythm is tuned to different values – varying from 1.25 Hz to 10 Hz. We have tested three model variants: two with exogenous theta rhythm (A and C, with and without preferred gamma speed respectively) and one with endogenous theta rhythm (variant A', for which we had also ideal onsets) on 44 sentences (randomly selected 2 sentences from each speaker). We also set the precision of the causal states of the gamma sequence to a higher value so that the dynamics of the endogenous slow oscillation is less distorted (for variant A'). The performance was significantly higher when the frequency of the theta neuron was < 10 Hz. When the preferred gamma speed was set by an endogenous theta rhythm, the best performance occurred for physiological theta values (2.5Hz to 8 Hz).

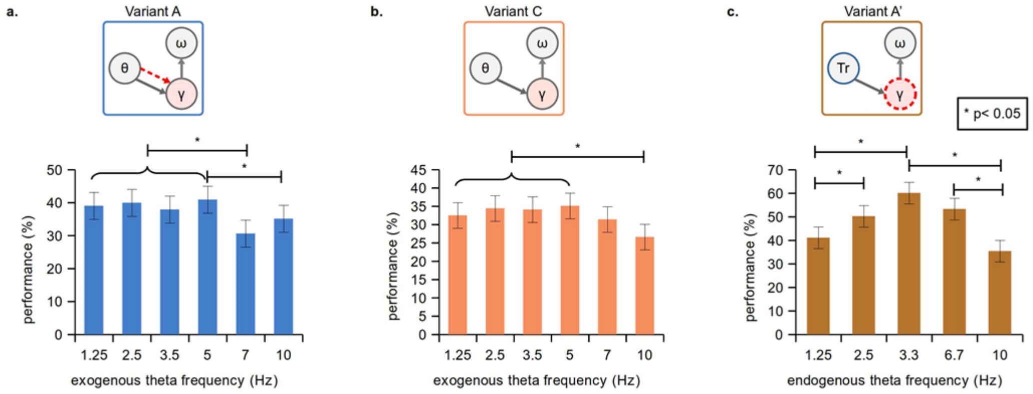

Figure 1: Model performance for different frequency values of slow oscillations. Performance of the model variants with an exogenous slow rhythm in the theta model (A and C, with and without preferred gamma speed respectively) and endogenous rhythm associated with the gamma sequence (variant A' with preferred gamma speed and ideal onsets). Bar plots illustrate the mean performance of each model variant for each condition (frequency of the slow oscillation on the x-axis). For each model variant, repeated measures ANOVA was used to compare the model's mean performance for each value of the theta frequency. p-values were corrected using the Tukey procedure (p-values less than 0.05 are considered statistically significant).

8. -At first glance more of a detail: why the very conservative false discovery rate threshold of q = 0.001? Figure 3 and Table S2 give the impression that the model variants differed quite profoundly from each other, but this also raises suspicion – why so vast differences between models, and would this have been different with more/more diverse speech materials as test cases?

We are thankful to the reviewer for raising these concerns about the statistical tests performed for evaluation of the model's performance. As already mentioned, we have increased the number of sentences in the data set from 30 to 220 and we have updated the statistical methods used for the model's performance evaluation. Moreover, we now report results based on more traditional, Bonferroni corrections for multiple companions.

REFERENCES:

1. Ermentrout, G. B. & Kopell, N. Parabolic Bursting in an Excitable System Coupled With a Slow Oscillation. *SIAM J. Appl. Math.* **46**, 233–253 (1986).

2. Hyafil, A., Fontolan, L., Kabdebon, C., Gutkin, B. & Giraud, A. L. Speech encoding by coupled cortical theta and gamma oscillations. *Elife* **4**, 1–45 (2015).

3. Davis, M. H. & Scharenborg, O. Speech perception by humans and machines. in *Speech Perception and Spoken Word Recognition* 181–203 (Psychology Press, 2016). doi:10.4324/9781315772110

Reviewers' Comments:

Reviewer #1:
Remarks to the Author:
The authors did a responsive revision and importantly they can now show that the model can process natural syllables. I have some remaining conceptual quibbles:

1. I think the use of the word "generative" in the Abstract is causing more confusion than clarity - yes the model 'generates' or parses syllables, but it is not generative in the sense that the word is often used in cognitive science and computational cognitive science. I think the authors mean the use of the word in the machine learning sense, but since terminology is rather inconsistent across these fields, it might just be simpler to leave it out.

2. The authors point out on line 269 that they assume syllables can appear in any order - this is patently unlike how natural language works, so this is a clear departure from the situation they are trying to model. On line 271 they argue that this is a 'more challenging situation' than when facing real speech statistics - I don't think this is fair. First, it mostly means their task is a poor approximation of the real problem the brain is solving, and second, it is not clear to me that matching stored representations in memory according to phonotactic rules is combinatorially easier or harder than not being constrained rule combinations if you have parallel processing, which they assume when using an associative memory algorithm. I would remove this claim.

3. One of the authors' main points is the important of the contribution of top-down information - yet they are again not modelling the complexity of the problem the brain solves - they don't include word-level or higher in formation or make use of prosodic structure or the full contents of the natural envelope. I think the limitation this models clearly faces needs to be discussed much more. It is a model of syllable tracking, but not of speech nor language processing.

Reviewer #2:
Remarks to the Author:
The authors have done a commendable job in appreciating my (i.e., both reviewers') concern(s).
I am overall content with the changes, and do think that the paper, with its level of detail and its attempts to model important aspects of the speech recognition/comprehension process in a neurobiologically (if not plausible then) thought-provocing way will be widely appreciated.

A remaining concern is that some of the results are of relatively weak/indecisive nature. To arbitrate between model architectures with (partly indecisive) p values is somewhat unusual, and not ideal. If the editors consider another round of revisions, more formal evidence on which model architecture "wins" not only w.r.t. performance but also taking into account model complexity would be desirable.

The theoretical conflation of frameworks predictive coding and neural oscillations (although the stimulus-driven model seems in fast to be winning?) was not entirely convincing to me, and the paper could surely use some severe text editing to make it into a more coherent whole (this latter being an editorial comment, rather).

In sum, I will have to leave it to the editors on whether this paper is a strong and compelling enough contribution for this journal.

Rebuttal

Dear editors and reviewers,

Thank you for giving us the opportunity of a second revision of our manuscript “Combining predictive coding and neural oscillations enables online syllable recognition in natural speech” (ID: NCOMMS-19-01489A).
Detailed responses to the reviewers are provided below.

Best regards,
Sevada Hovsepyan

Reviewer #1 (Remarks to the Author):

The authors did a responsive revision and importantly they can now show that the model can process natural syllables. I have some remaining conceptual quibbles:

1. I think the use of the word "generative" in the Abstract is causing more confusion than clarity - yes the model 'generates' or parses syllables, but it is not generative in the sense that the word is often used in cognitive science and computational cognitive science. I think the authors mean the use of the word in the machine learning sense, but since terminology is rather inconsistent across these fields, it might just be simpler to leave it out.

We thank the reviewer for the suggestion and changed the phrase “generative model” in the abstract with “computational model”.

2. The authors point out on line 269 that they assume syllables can appear in any order - this is patently unlike how natural language works, so this is a clear departure from the situation they are trying to model. On line 271 they argue that this is a 'more challenging situation' than when facing real speech statistics - I don't think this is fair. First, it mostly means their task is a poor approximation of the real problem the brain is solving, and second, it is not clear to me that matching stored representations in memory according to phonotactic rules is combinatorially easier or harder than not being constrained rule combinations if you have parallel processing, which they assume when using an associative memory algorithm. I would remove this claim.

This is a fair point. However, our claim was based on the notion that using real speech statistics instead of assuming combinatorial freedom between syllables would narrow down the search and improve the model performance. As shown in the Figure 1 below

this is already the case when we decrease the number of syllables in the sentence. All versions of the model perform better with short than long sentences, confirming it is easier for the model(s) to pick the correct syllables from fewer choices (as would be the case if we used natural speech statistics).
As our formulation might indeed be confusing we removed it from the manuscript.

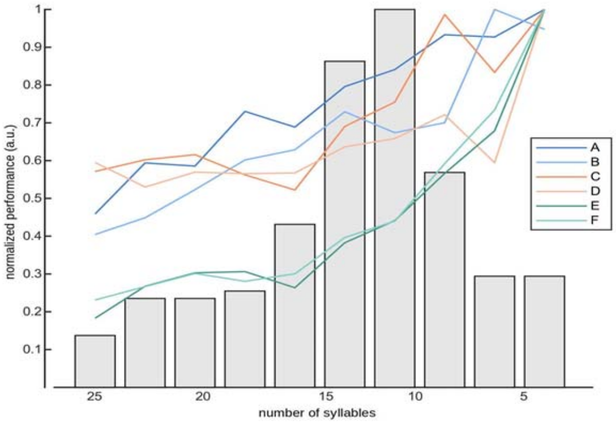

Figure 1: The histogram represents syllable number distribution across sentences (normalized so that the highest value is equal to 1). Each colored line represents the performance of the corresponding model variant (color-coded as in Figure 3 in the manuscript) depending on the number of syllables in the sentence (the highest performance for each variant is normalized to 1 to better visualize the decrease in performance with increased number of potential syllables).

3. One of the authors' main points is the important of the contribution of top-down information - yet they are again not modelling the complexity of the problem the brain solves - they don't include word-level or higher information or make use of prosodic structure or the full contents of the natural envelope. I think the limitation this models clearly faces needs to be discussed much more. It is a model of syllable tracking, but not of speech nor language processing.

The goal of the current study is to propose a model of “*on-line”* syllable parsing and identification from natural sentences, and as argued in the manuscript, this is an essential step toward natural speech processing. Lexico-semantic top-down processing is beyond the scope of this study, which intends to explore the possible advantage of combining *generic mechanisms*, namely neural oscillatory activity as temporal constraints on top-down/bottom-up informational flows.
We certainly agree that speech “perception” is a more complex process than what the model describes and that “content-related top-down” will have to be taken into account in possible follow-ups of this work. We have further clarified this point in the discussion section (lines 273 - 287).

Reviewer #2 (Remarks to the Author):

The authors have done a commendable job in appreciating my (i.e., both reviewers') concern(s). I am overall content with the changes, and do think that the paper, with its level of detail and its attempts to model important aspects of the speech recognition/comprehension process in a neurobiologically (if not plausible then) thought-provocing way will be widely appreciated.

A remaining concern is that some of the results are of relatively weak/indecisive nature. To arbitrate between model architectures with (partly indecisive) p values is somewhat unusual, and not ideal. If the editors consider another round of revisions, more formal evidence on which model architecture "wins" not only w.r.t. performance but also taking into account model complexity would be desirable.

We thank the reviewer for this remark. We initially compared different model architectures while keeping the exact same number of components (Figure 3), which indeed led to weakly contrasted results. As an alternative, rather than just turning off components in the different model variants, we removed them (e.g. slow amplitude modulation and theta module from variant B). The new simulation results (Figure 2 below) show that the model variants with removed components (B', D', F') have the same performance as variants with switched off components (B, D, F) in terms of correctly identified syllables.

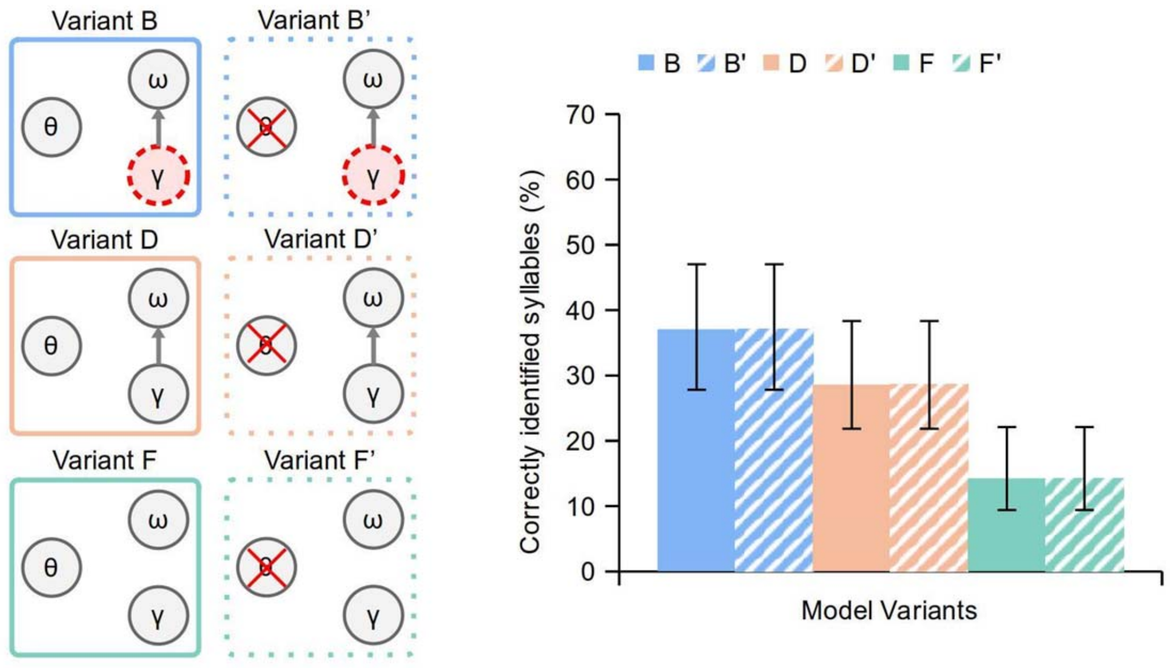

Figure 2: Comparison of the model variants with switched off components as used in the manuscript (B, D, F) versus model configurations where the corresponding components were removed (red cross on the theta module, B', D', F').

To compare models with different complexity, we used the Bayesian information criterion[1] (BIC) that takes into account both accuracy and complexity. Variant A had the highest BIC and hence the best accuracy/complexity trade-off. As the BIC was overall higher for those models where modules were removed (B', D' and F') rather than turned-off (B, D and F), we concluded that removing "extra" modules reduced the model complexity without impacting its performance. We therefore only present results with removed components in the revised manuscript (Figure 3, below).

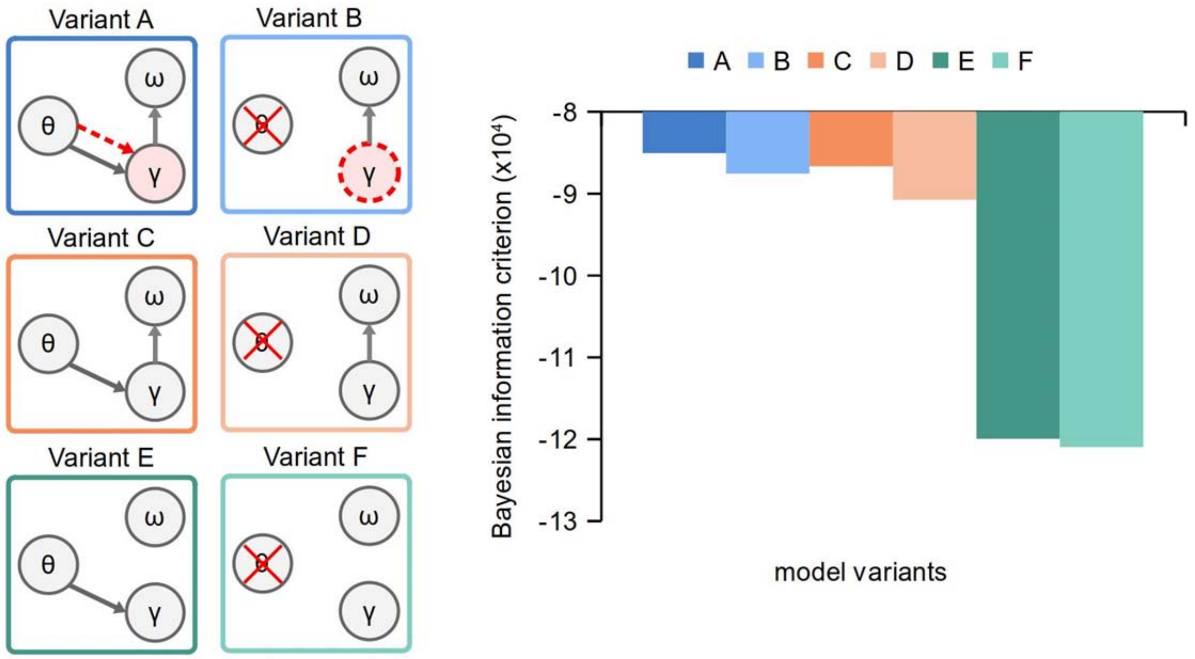

Figure 3: The Bayesian information criterion value (across all sentences) for each model variant (B, D, F here correspond to variants with removed components).

The theoretical conflation of frameworks predictive coding and neural oscillations (although the stimulus-driven model seems in fast to be winning?) was not entirely convincing to me, and the paper could surely use some severe text editing to make it into a more coherent whole (this latter being an editorial comment, rather).

In sum, I will have to leave it to the editors on whether this paper is a strong and compelling enough contribution for this journal.

Studies that connect predictive coding with neural oscillations mostly assume neural oscillations as "channels" to transmit information across cortical hierarchical levels,

assigning distinct frequency channels to top-down and bottom-up information passing[2–4]. This study approaches the issue from a different (and to our knowledge, novel) perspective: that is to explore the possible advantages of neural oscillations coupling for bi-directional informational passing during inferential processes. We have modified the 'conclusions' part in the 'Discussion' section and hope that the originality of the approach now clearly appears in the manuscript.

References

1. Schwarz, G. Estimating the Dimension of a Model. *Ann. Stat.* **6**, 461–464 (1978).
2. Fontolan, L., Morillon, B., Liegeois-Chauvel, C. & Giraud, A. L. The contribution of frequency-specific activity to hierarchical information processing in the human auditory cortex. *Nat. Commun.* **5**, 4694 (2014).
3. Sedley, W. *et al.* Neural signatures of perceptual inference. *Elife* **5**, e11476 (2016).
4. Chao, Z. C., Takaura, K., Wang, L., Fujii, N. & Dehaene, S. Large-Scale Cortical Networks for Hierarchical Prediction and Prediction Error in the Primate Brain. *Neuron* **100**, 1252-1266.e3 (2018).

Reviewers' Comments:

Reviewer #1:
Remarks to the Author:
I am happy with the revision.

Reviewer #2:
Remarks to the Author:
My apologies for the pandemic-related delay to all parties involved. The manuscript has gained considerably in clarity and potential impact through this round of reviews, and I have no intention of standing in the way of these results making their way into publication.

Jonas Obleser, University of Lübeck
