# Supplementary material for: Combining predictive coding and neural oscillations enables online syllable recognition in natural speech
Source: Nat Commun. 2020 Jun 19;11:3117. doi: 10.1038/s41467-020-16956-5 (PMC7305192; doi:10.1038/s41467-020-16956-5)
Supplement: Supplementary file 2 — Peer Review File [file 41467_2020_16956_MOESM2_ESM.pdf]

Supplementary information

Combining predictive coding and neural oscillations enables online syllable recognition in natural speech

Hovsepyan et al.

Supplementary Figures

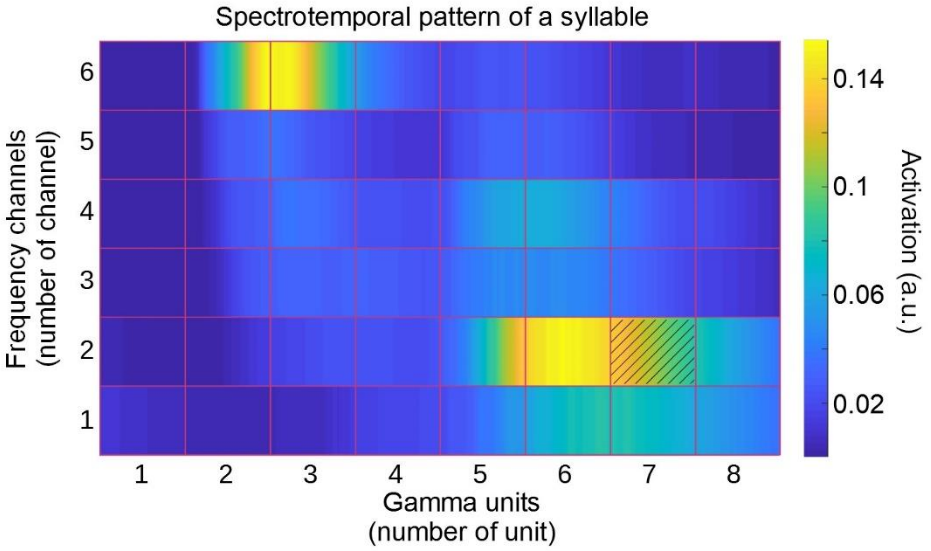

Supplementary Figure 1. Extraction of spectrotemporal patterns. The figure illustrates how the spectrotemporal pattern ($\mathrm{ST}_{f\gamma\varpi}$) of each syllable was calculated. We divide each syllable into 8 bins of equal duration. As we have six frequency channels and eight gamma units per syllable, we created 6x8 matrices where each entry corresponds to the average amplitude of the associated frequency channel over the duration of each of the 8 temporal bins. For example, the entry $\mathrm{ST}_{27\varpi}$ (f=2 and γ=7), the dashed entry on the figure, represents the average activation of the second frequency band within the seventh gamma cycle.

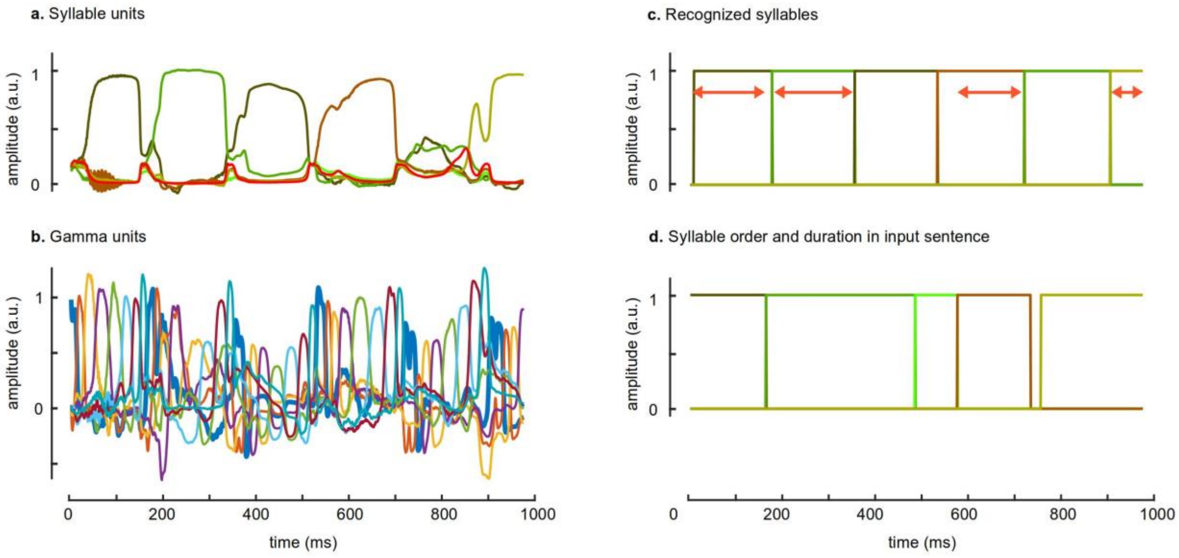

Supplementary Figure 2: Dynamics of syllable and gamma units and performance evaluation. Panels **a** and **b** show the dynamics of syllable and gamma units for an example sentence; "Brakes shrieked behind us". For the syllable units, each colour corresponds to a syllable (except the bright red line that is reserved for a silent unit - a unit that represents "silence" in the input). For the gamma units, each colour corresponds to a specific gamma unit in the sequence (blue corresponding to the first unit and teal corresponding to the last (8-th) unit). Panels on the right side of the plot illustrate order and duration of each syllable in the input sentence (panel **d**) and the model's output (panel **c**) based on the dynamics of the syllable units (panel **a**). We select the syllable unit that has the highest average activation between two consecutive gamma-1 unit activations (highlighted blue unit in panel **b**) as those represent when the model started a new gamma sequence for a new syllable. Therefore, we quantify how long the selected syllable units correspond to the syllable in the input (red arrowed lines in panel **c**). For this particular sentence (with a duration of 975 ms) the duration of the overlap was 582ms; hence model performance was 59.7% (100 * 582/975).

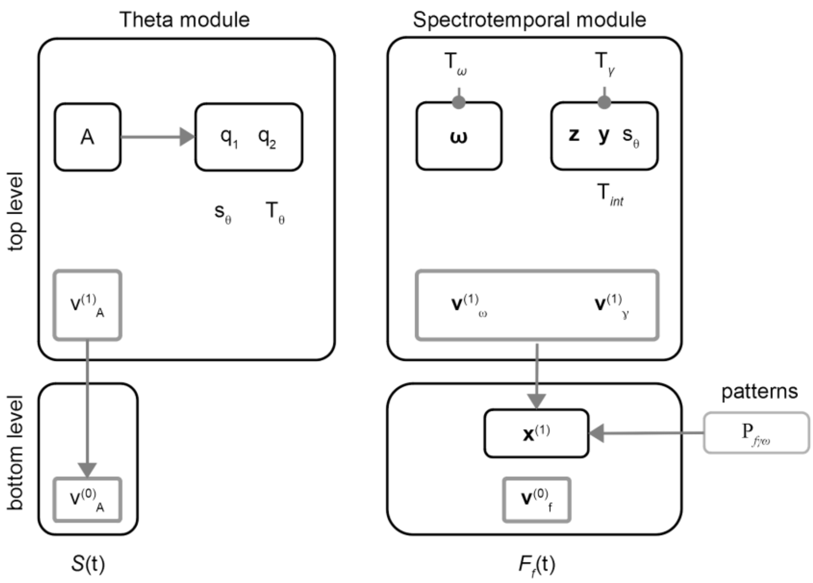

Supplementary Figure 3. Schematics of the model. The figure shows all the variables in the model. Black boxes include the hidden states of each level, whereas grey boxes correspond to the information that each level passes to the level below. The top-level sends information about the slow amplitude modulation $\upsilon_A^{(1)}$ and the dynamics of the syllable $\upsilon_\omega^{(1)}$ and gamma units $\upsilon_\gamma^{(1)}$. The output of the first level ($\upsilon_A^{(0)}$ for the slow amplitude modulation and $\upsilon_f^{(0)}$ for the frequency channels) is then compared with the input signal (*S(t)* and $F_f(t)$ respectively).

Supplementary Tables

Supplementary Table 1.

Model variant	A	B	C	D	E	F
A		0.765	8.41e-11	1.16e-11	9.93e-35	7.26e-35
B			3.35e-9	9.8e-12	2.68e-35	1.14e-35
C				0.1733	6.19e-31	5.62e-31
D					6.84e-28	4.59e-28
E						0.8177
F						

p-values for pairwise comparisons. For pairwise comparisons (overall N=15), the Wilcoxon signed-rank test was used. All differences, except A vs. B, C vs. D and E vs. F, are highly significant with p < 1e-7/N (Bonferroni correction).

Supplementary Table 2.

Model variant	A	B	C	D	E	F
A		2520	1607	5721	34834	36035
B			-912	3201	32314	33516
C				4114	33227	34428
D					29113	30314
E						1201
F						

Bayesian Information Criterion. Each row shows how much the BIC value of the corresponding model variant is bigger/smaller than the BIC value of other variants.
